# Oil spill risk analysis for the NEOM shoreline

**DOI:** 10.1038/s41598-024-57048-4

**Published:** 2024-03-19

**Authors:** H. V. R. Mittal, Mohamad Abed El Rahman Hammoud, Ana K. Carrasco, Ibrahim Hoteit, Omar M. Knio

**Affiliations:** 1 Computer, Electrical and Mathematical Sciences and Engineering Division, King Abdullah University of Science and Technology (KAUST), 23955-6900 Thuwal, Saudi Arabia; 2 Physical Science and Engineering Division, King Abdullah University of Science and Technology (KAUST), 23955-6900 Thuwal, Saudi Arabia; 3 Department of Mathematics, Indian Institute of Technology Ropar, Rupnagar, Punjab 140001 India

**Keywords:** The Red Sea, NEOM, Oil spill, Risk analysis, MOHID, Environmental impact, Computational science, Physical oceanography

## Abstract

A risk analysis is conducted considering an array of release sources located around the NEOM shoreline. The sources are selected close to the coast and in neighboring regions of high marine traffic. The evolution of oil spills released by these sources is simulated using the MOHID model, driven by validated, high-resolution met-ocean fields of the Red Sea. For each source, simulations are conducted over a 4-week period, starting from first, tenth and twentieth days of each month, covering five consecutive years. A total of 180 simulations are thus conducted for each source location, adequately reflecting the variability of met-ocean conditions in the region. The risk associated with each source is described in terms of amount of oil beached, and by the time required for the spilled oil to reach the NEOM coast, extending from the Gulf of Aqaba in the North to Duba in the South. To further characterize the impact of individual sources, a finer analysis is performed by segmenting the NEOM shoreline, based on important coastal development and installation sites. For each subregion, source and release event considered, a histogram of the amount of volume beached is generated, also classifying individual events in terms of the corresponding arrival times. In addition, for each subregion considered, an inverse analysis is conducted to identify regions of dependence of the cumulative risk, estimated using the collection of all sources and events considered. The transport of oil around the NEOM shorelines is promoted by chaotic circulations and northwest winds in summer, and a dominant cyclonic eddy in winter. Hence, spills originating from release sources located close to the NEOM shorelines are characterized by large monthly variations in arrival times, ranging from less than a week to more than 2 weeks. Similarly, large variations in the volume fraction of beached oil, ranging from less then 50% to more than 80% are reported. The results of this study provide key information regarding the location of dominant oil spill risk sources, the severity of the potential release events, as well as the time frames within which mitigation actions may need to deployed.

## Introduction

**Figure 1:**
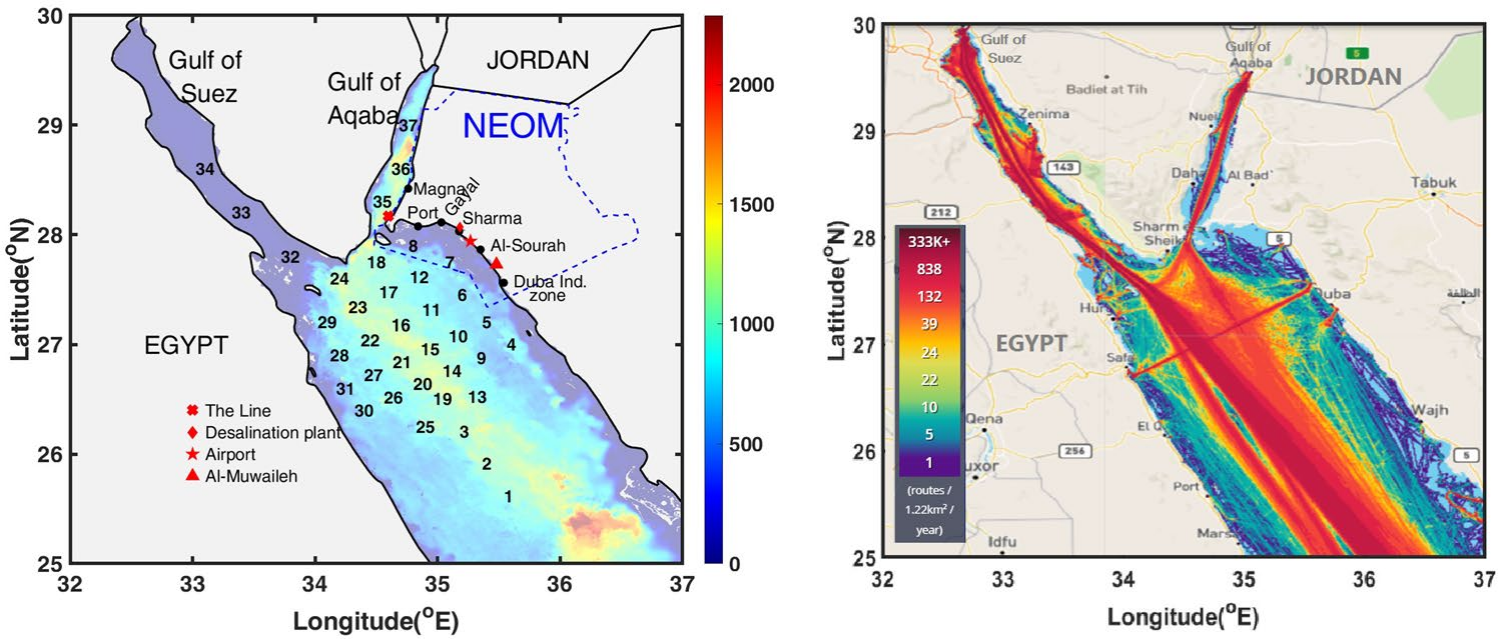
(Left) General map of the northern Red Sea region illustrating key installations along the NEOM coastlines as well as the source locations chosen for spill simulations as well as the bathymetry of the northern Red Sea colored by depth in meters, and (Right) Contours exhibiting the marine traffic density, as the number of routes per 1.22 km^2^ averaged over a year^2^.

NEOM is a futuristic city being developed in the Tabuk province^1^, Kingdom of Saudi Arabia. It is situated in the north western part of the Kingdom with miles of Red Sea coastlines. At its northernmost point, it is just 50 km from the Jordanian port of Aqaba. NEOM development plans include establishing modern manufacturing facilities, industrial research and development, in addition to a hydrogen plant, a desalination plant and an international airport (see Fig. 1). Tourism facilities are also being developed along its coastal environment hosting a diverse marine wildlife and coral reserves^1^.

With an estimated 6.2 million barrels per day of crude oil and refined petroleum products transported through its main shipping lanes in 2018^3^, the Red Sea is one of the most active waterways in the world^4^. This poses a risk of accidental oil spills that may contribute to marine pollution, disrupting desalination operations, and consequently causing severe economic losses and irreversible damages to the environment^4–7^. Therefore a comprehensive analysis of risk from accidental oil spill releases on coastal Red Sea regions is of paramount importance, particularly to minimize potential impact to both the environment and industrial activities, and to plan emergency response and mitigation efforts in case of an accident.

Several studies assessed the risk of oil spill accidents for different regions around the world. These encompassed the Mediterranean sea^8–12^, the southern Adriatic and the northern Ionian (SANI) sea^13^, Canadian waters^14^, Caribbean sea^15^, Sicily coasts^16^ and Bay of Bengal^17^. A few studies have investigated the risk of oil spills on specific regions of the Red Sea, namely pertinent to the Egyptian coastlines^18^, the Bashayer shorelines^19^ and the Saudi Arabian-Yemeni coastlines^7^. Periáñez^20^ presented a Lagrangian model for the whole Red Sea. Mittal et al.^4^ provided a broad assessment of oil spill hazards for the whole Red Sea, stemming from its main shipping lane along the longitudinal axis. Pertinent to the risk analysis of oil spills for the NEOM shoreline, a study is still lacking, where existing studies in the literature that focus on NEOM encompass atmospheric conditions and air quality assessment^21^, geological assessment^22,23^ and wind energy assessment^24^ only.

This study is part of an effort aimed at developing a fundamental understanding of the risk associated by possible oil release sources on the NEOM coastline, and consequently establishing a knowledge base that can assist in the design of efficient strategies safeguard its coastal environment from accidental oil spills. Specifically, a hazard analysis is conducted considering an array of 37 potential release locations, distanced approximately 30 km apart, located around the NEOM coastline in the regions of high marine traffic (see Fig. 1). The risk associated with each source is described by the amount of oil beached following the initial release, and by the time required for the spilled oil to reach the NEOM coast. The evolution of the oil spill is simulated using the MOHID oil spill model^25–28^. The model enables realistic, three-dimensional simulations of oil trajectories, accounting for weathering phenomena such as evaporation, dispersion, sedimentation, dissolution, and emulsification. Extensively-validated, high-resolution met-ocean fields^29^ of the Red Sea are used to drive the oil spill model. For each release source, simulations are conducted over a 28-day period, starting from the first, tenth and twentieth days of each month, covering five consecutive years ranging from 2013 to 2017. A total of 180 simulations are thus conducted for each source, adequately reflecting the variability of met-ocean conditions in the region. In addition to characterizing the impact of individual sources, the simulation results are analyzed by segmenting the NEOM shoreline, extending from the Gulf of Aqaba in the North to Duba in the South, based on important coastal developments and installations. For each subregion, an inverse analysis is finally conducted to identify regions of dependence of the cumulative risk estimated using the collection of sources considered.

## Methods and data

### Red Sea met-ocean reanalysis

Met-ocean data are extracted from an extensively-validated reanalysis of the circulation in the Red Sea. For more details related to the validation exercises, readers are referred to recent works by Hoteit et al.^29^, Vankayalapati et al.^30^, Wang et al.^31,32^, Krokos et al.^33^, Zhan et al.^34^, Yao and Hoteit^35^. The simulated fields have been shown to suitably describe the general oceanic and atmospheric circulations of the Red Sea at the highest available resolution^29–38^. The zonal and meridional winds were fetched from a 5 km regional atmospheric reanalysis generated using the Weather Research Forecasting (WRF) model assimilating all available regional observations^37,38^. WRF initial and boundary conditions were acquired from the European Centre for Medium-Range Weather Forecasts (ECMWF) reanalysis Interim data^39^ (ERA-I). The wave conditions^40^ in the Red Sea were reconstructed using the WAVEWATCH III (WWIII) model forced with the aforementioned high-resolution WRF reanalysis winds^41^ on a uniform grid of 1 km resolution.

The MIT general circulation model (MITgcm^42^) was implemented to simulate the 3D ocean currents on a grid with 1-km resolution in horizontal planes and 50 vertical layers. The model was forced using the aforementioned high-resolution WRF reanalysis fields and the Copernicus Marine Service (CMS) global ocean reanalysis fields^43^ across the open-boundary in the Gulf of Aden at a 6 hourly and 24 hourly temporal frequency, respectively. The resulting MITgcm outputs for the Red Sea have been extensively employed to analyze the general and over-turning circulations^44,45^, internal/baroclinic tides^46^, mesoscale eddies characteristics^34^, deep-water formation events^35^, temperature and salinity budgets^47^ as well as the chlorophyll variability^48^. We refer readers to^29^ for a more detailed description of the met-ocean conditions.

### Northern Red Sea circulation

Mesoscale eddies^49,50^ play a dominant role in pollutant transport in the northern Red Sea region. A typical cyclonic eddy dominates the circulation during the winter season, and is characterized by a rotational velocity that are generally larger than that of the background flow^4^. These eddies tend to become more energetic during winter months following the development of intense baroclinic instabilities^49,51^, and they represent the dominant structures except for some strong semi-permanent wind-driven gyres that occur in summer^52^.

The high mountain ranges on both sides of the Red Sea forces the wind to blow along its axis^53^. During summer seasons, from April till October, a northwest (NW) wind blows along the whole length of The Red sea, with speeds close to 10 ms^-1^, and frequently exceeds 15 ms^-1^^41^. During winter, the same northerly wind dominates over the northern part of the basin. The narrower valleys along the eastern coasts of the Red Sea also creates westward blowing jets in the northern part and generally lasts for 2–3 days with a maximum speed up to 15 ms^-1^. The wave variability in the Red Sea is naturally associated with the dominant regional wind regimes^53^. Despite the moderate winds, the prolonged duration and long fetch along the whole basin may generate waves as high as 3.5 m. During the summer months, the northwesterly winds prevailing over the whole Red Sea generate mean wave heights of 1 m–1.5 m in the north^53,54^, throughout the year.

### Oil spill model

The MOHID oil spill model was adopted to simulate the instantaneous release of oil and its evolution from fixed sources in the northern Red Sea. It relies on a Lagrangian formalism that considers oil as a collection of Lagrangian particles and associates to each particle oil properties and a location^55,56^. The Lagrangian particles are transported using the met-ocean conditions, and their properties are updated by solving empirical models describing physico-chemical transformations of oil. Typically, these weathering processes result in changes in oil’s physical properties and also impact the oil slick’s geometry. The grid cells in the bathymetry file used in simulations are categorized coastal cells and sea cells. An oil particle that reached a coastal cell is considered to be beached. In the present study, dissolution and sedimentation processes were not considered, thus eliminating their effect on the oil mass balance. However, evaporation, dispersion and emulsification were accounted for. Specifically, evaporation processes are described by the algorithms of Stiver and Mackay^57^ which are mainly based on oil properties and area of the slick. The fraction of sea surface capped by breaking waves (white caps) per unit time is computed following Delvigne and Sweeney^58^ whereas emulsification processes are represented using the algorithms by Mackay et al.^59^. Entrainment of surface oil due to breaking waves significantly influences the spatial and temporal evolution of oil particles on the sea surface. In our simulations, the entrainment rate, or the amount of oil dispersed in the water column, is computed following the methodology of Mackay et al.^59^ Spreading is computed following the empirical thickness gradient model of Fernandes^60^. This model is based on assumption that the thickness gradient generates a spreading force in the direction of smaller thickness. The Stokes drift velocity is calculated using the algorithm of Daniel et al.^61^. The corresponding velocities obtained by these algorithms are added to the horizontal particle velocities interpolated from the met-ocean fields. Finally, the influence of surface winds on the motion and deformation of the oil slick was incorporated using a wind coefficient of $3\%$^62^.

Our study is focused on performing a source risk analysis, considering a wide array of potential release sources in the northern Red Sea. Consequently, we have adopted a conservative approach based on considering that oil particles are beached as soon as they reach a coastal cell. Whereas this approach ignores important mechanisms that govern the ultimate fate of the spill, such as the formation of oil particle aggregates^63,64^ and oil bio-degradation^65^, the resulting compromise enables to avoid resorting to detailed resolution of the hydrodynamics in the near-shore region and consequently affords the efficiency needed to consider a wide array of potential release sources.

### Experimental setup

As briefly discussed below, the present study adapts the setup presented in^4,66^ to the region surrounding NEOM. The computational domain covers the northern Red Sea region, extending across the longitudes $32^\circ$ to $37^\circ$ and latitudes $25^\circ$ to $30^\circ$ and up to a depth of approximately 2746 m . The bathymetry features a Cartesian grid that consists of vertical layers from the depth of the domain to the bottom layer where each layer has a fixed depth. The resulting computational mesh is uniform in horizontal planes and non-uniform in the vertical direction. It uses 500 equally-spaced nodes along the longitudinal axis, 500 equally-spaced nodes along the latitudinal axis, and 50 layers in the vertical direction. The horizontal grid resolution is approximately 1 km.

From the met-ocean fields outlined above, the 3D ocean currents, surface winds, wave height and wave period from the years 2013 till 2017 were extracted and used an inputs to drive MOHID. 2000 oil particles are released from each source comprising a spill volume of 10,000 $m^3$. The particle advection velocities are obtained using bilinear interpolation of the met-ocean fields at the particle locations. The Arabian crude oil with a specific gravity of 27.4^∘^ API, pourpoint of − 28^∘^ and dynamic viscosity of 1 cP is chosen as an assumptive release oil for the simulations. The horizontal and vertical turbulent diffusivities are set to 5 m^2^/s and 0.001 m^2^/s, respectively. The values of these oil parameters were selected following Hammoud et al.^66^, where a global sensitivity analysis with respect to a wide range of oil parameters was conducted. Because oil processes vary over short time scales, weathering processes were simulated using a time step of size 60 s. On the other hand, to minimize the computational cost, the Lagrangian particle transport model used a time step of size 3600 s. In all cases, an explicit first-order time integration scheme is adopted.

### Risk quantification

The risks of individual oil spill sources are quantified in terms the arrival times of oil particles, and the volume fractions of oil beached on the NEOM shorelines. The arrival times represent the time elapsed from the moment of their release for individual oil particles to reach the NEOM shorelines. For each source, the volume fractions reflect the ratio of oil volume beached to the volume initially released. The arrival times are divided into four classes, namely $< 7$ days, 7–14 days, 14–28 days, and $>28$ days (as surrogate for no arrival during the simulation period). Similarly, the volume beached are divided into four classes, namely $>50 \%$ of the initial release, 25–50%, $<25 \%$, and $0 \%$ (when no oil is beached). The results are illustrated using pie charts that depict the frequencies of the classes considered. When generated for individual months of the year, the charts represent the outcome of fifteen experiments, as three simulations per month are performed for the five consecutive years investigated.

A finer analysis is also conducted where, instead of considering the entire NEOM coastline, smaller segments (approximately 25-km wide) are considered around specific sites, namely The Line, Duba, Sharma, Gayal and Magna. For each site, a histogram of the volume fraction is generated showing, for each source and release event considered, the amount of volume beached classified (using colors) in terms of the corresponding arrival time class. The histograms provide key information regarding the severity of the potential release event, and the time frame within which mitigation actions need to be deployed to minimize the impact on coastal areas.

Finally, an aggregate probability of volume beached along a given shoreline ($p_{i}$) is computed as:

1 $$\begin{aligned} p_i = \frac{\sum _{j=1}^{15} {\mathscr {V}}_{i, j}}{\sum _{k=1}^{37} \sum _{j=1}^{15}{\mathscr {V}}_{k, j}}, \end{aligned}$$

where ${\mathscr {V}}_{i,j}$ is the fraction of volume beached from release location *i* for event *j*, such that the event *j* is an enumeration on the release times. The aggregate probability of volume beached measures the contribution of a given release source with respect to all the release sources. This metric allows contrasting sources by ranking release source based on their likely impact on the NEOM shoreline.

**Figure 2:**
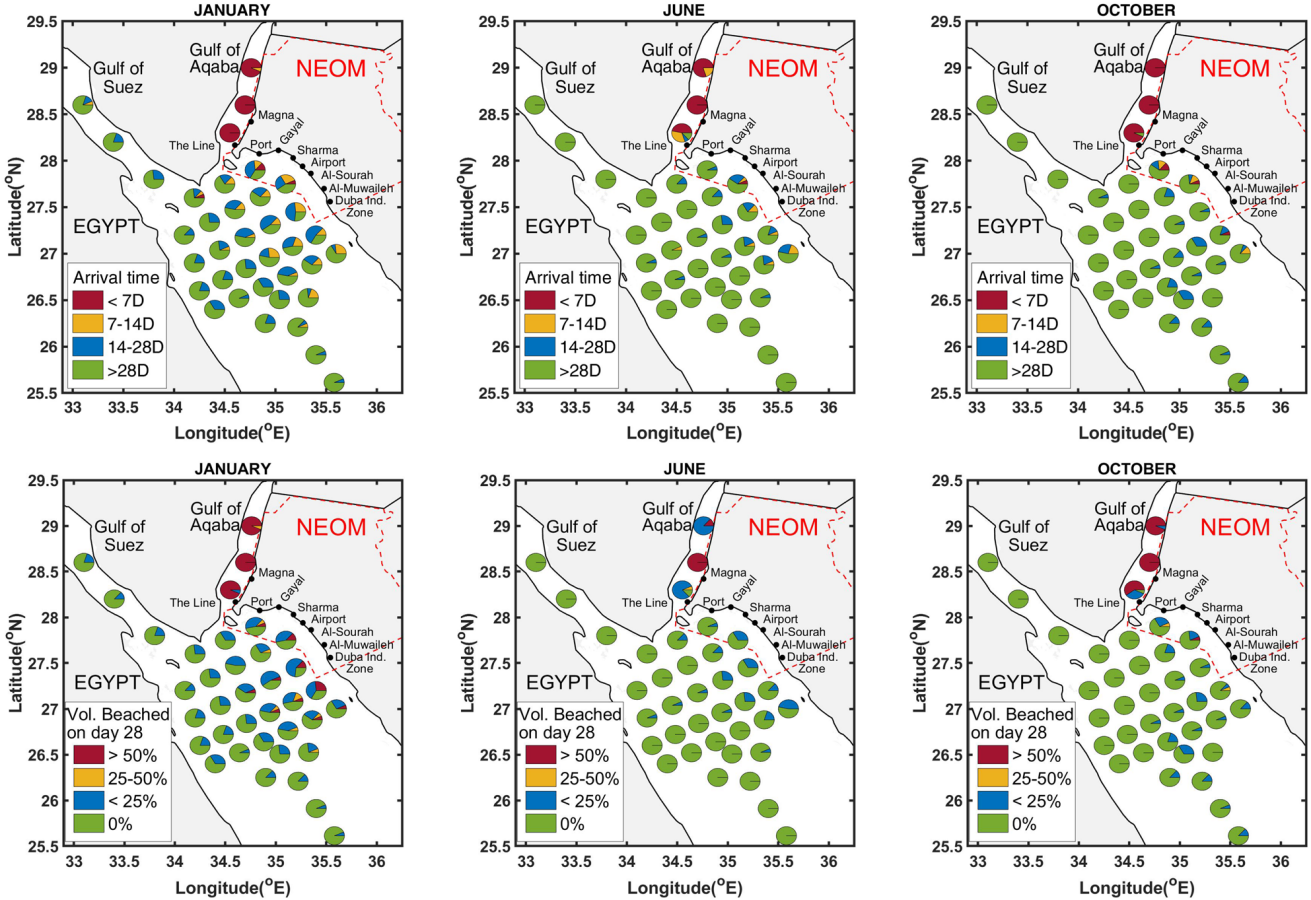
Pie charts centered at each release source, representing the corresponding arrival times (top row) and the volume fractions of beached oil particles (bottow row).

**Figure 3:**
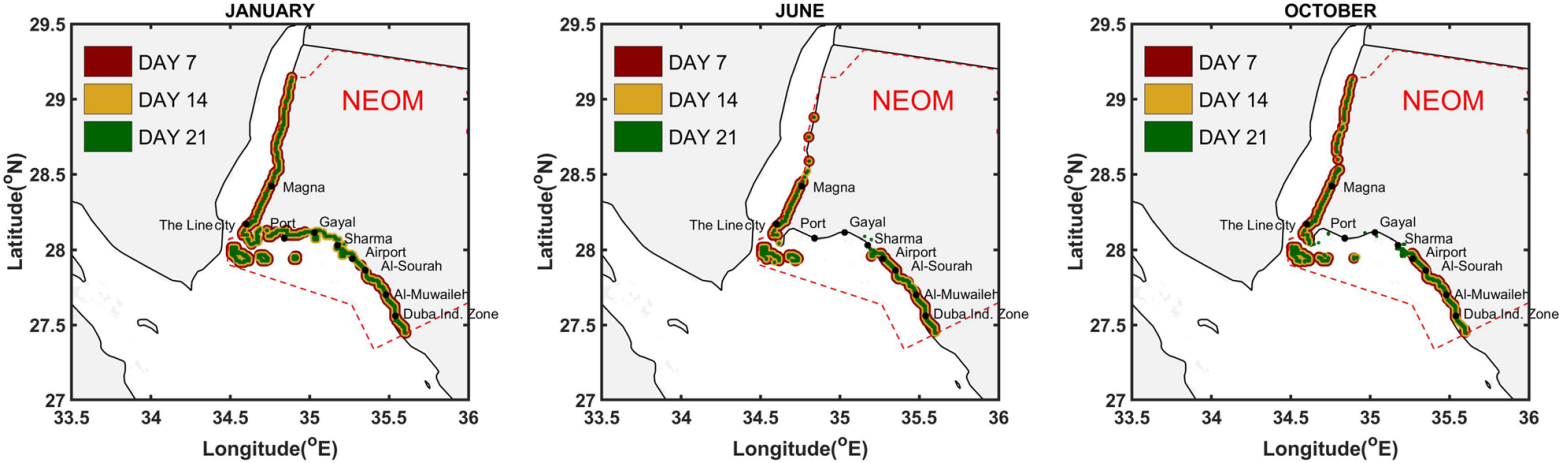
Regions of the Neom shoreline affected by beaching, for 7, 14 and 21 days after the onset of the spill. Particles originating from all release sources are used to generate the contours. Plots are generated for release events occurring during the January, June and October months, as indicated.

## Results and discussion

### Risk analysis for the NEOM shoreline

Figure 2 and Supplementary Figures S1–S2 illustrate pie charts representing the impact of fifteen release events occurring during the months from January to December. The pie charts depict, for each release source, the time needed by the oil particles to reach the NEOM shoreline as well as the volume fraction of oil beached at the end of the simulation period. Figure 3 and Supplementary Figure S3 depict the region of the NEOM shoreline affected by beached oil particles, 7, 14 and 21 days following the release. Particles originating from all release sources are used to generate these contours, thus illustrating the aggregate risk. Release events originating during the months of January, June, and October are used for this purpose.

Figure 2 and Supplementary Figure S1 indicate that spills originating from sources $S_{35}-S_{37}$, which are located in the narrow Gulf of Aqaba and thus close to the shorelines, are characterized by short arrival times. Within 1 week from the onset of the spill, entire segments of NEOM shoreline adjoining the Gulf of Aqaba are generally impacted; this occurs for all scenarios except for a few releases occurring during the summer months. In the summer months, the prevailing southwards currents in the Gulf of Aqaba tend to push the oil slicks towards the Tiran and Sanfir islands. Therefore, some segments of shorelines, located north of Magna city, may be shielded. Within 1 week from the time of the spill, over 50% of the volume of oil released by sources ($S_{35}-S_{37}$) may generally beach on the NEOM shore. This occurs over the whole year except for the month of June. In June, the volume fraction of oil released from source $S_{35}$ that beaches on the NEOM shore is less than $25 \%$ by the end of the first week, but may rise to around $50 \%$ by the end of the third week following the onset of the spill.

The arrival times of oil particles originating from most of the sources in $S_{4}-S_{8}$ are less than 1 week during the whole year except during the months of June (except $S_{7}$), September and October. The volume fractions of oil beached originating from sources $S_{4}-S_{8}$ are less than $25 \%$ by the end of the first week, but may rise to greater than $50 \%$ within 2 weeks after the onset of the spill, during Jan–May, July and August. These volume fractions are seen to exceed 50% by the end of the first week of the onset during the months of November and December. This transport of spilled oil towards the NEOM shorelines is promoted by a cyclonic eddy that dominates the circulation in the Northern Red Sea region during the winter seasons^4^.

For the majority of release sources $S_{19}-S_{29}$, located in the open waters and close to the Egyptian coast, the arrival times fall in the interval of 2–3 weeks from the onset, for the months of November–March and July. By the end of third week after the onset of the spill, the volume fractions of oil originating from these sources remain below $25 \%$. During the remaining months, only a few of the sources $S_{19}-S_{29}$ could impact the NEOM shorelines. Furthermore, the volume fraction of oil beached is less than $25 \%$, with relatively longer arrival times of around 4 weeks or no beaching in some scenarios.

For sources $S_{32}-S_{34}$, which are located in the Gulf of Suez, a measurable impact on the NEOM shoreline is only observed during the months of January–May and July. Beaching of oil originating from $S_{32}$ is recorded after week one during February, within 1–2 weeks in March and in May, 2–3 weeks in January and July. Oil released from $S_{33}$ impacts the NEOM shorelines within 2–4 weeks in May and from January–March. For $S_{33}$, the arrival times fall within 2–3 weeks in January and 3–4 weeks in April and May. The volume fraction of oil released by sources $S_{32}-S_{34}$ and beached on the NEOM shore remains less than 25% by the end of the fourth week, following the onset of the spill.

Figure 3 shows that the NEOM shoreline extending from The Line in the north to Duba in the South is impacted in its entirety during January to May, but during June to December some segments are not significantly impacted. Specifically, by the end of the third week after the onset of the spill, beaching on the shoreline between Sharma and The Line is not predicted during June and from September to October. Additionally, beaching on the shoreline between The Port of NEOM and The Line is not observed from May to September. The energetic meso- and submeso-scale circulations and northwesterly winds in the northern Red Sea region tend to split the oil slicks into different fragments^4^. These fragments are then transported in the opposite directions, towards both the Egyptian and Saudi Arabian shorelines, thereby sparing some segments between The Line and Sharma from beached oil during the months from June–December.

**Figure 4:**
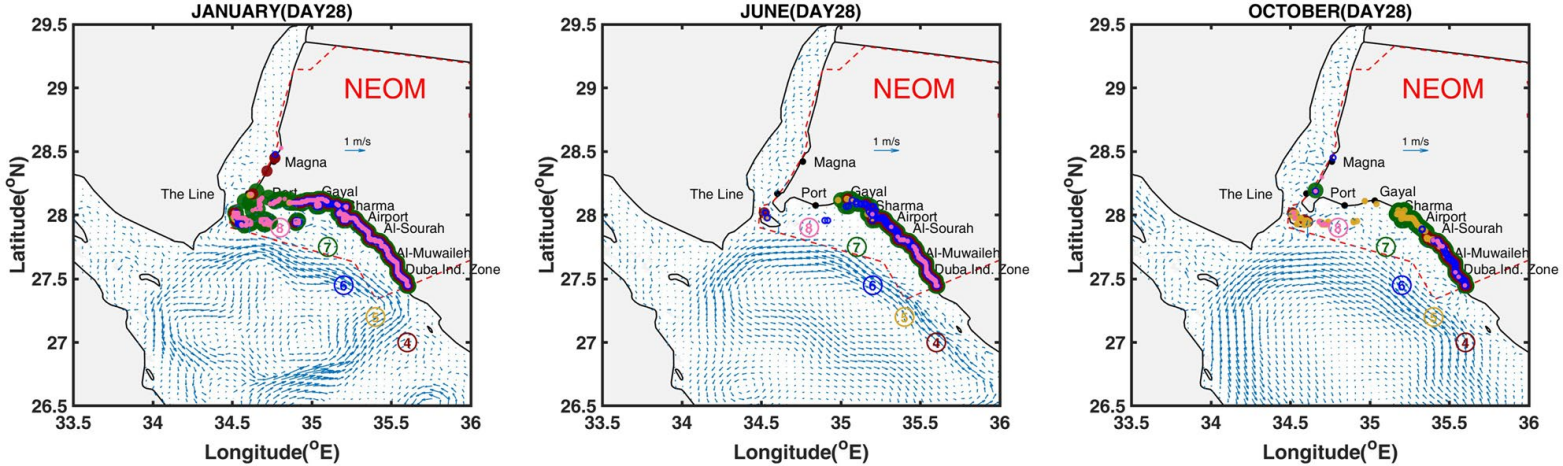
Regions of the Neom shoreline affected by beaching at the end of the 28-day simulation period. The contributions of selected sources are isolated using different color scheme for the individual sources, as indicated. Plots are generated for release events occurring during the January, June and October months, as identified by the titles.

Figure 4 and Supplementary Figure S4 isolate the contributions of release sources $S_{4}-S_{8}$ which lie inside the NEOM boundary and are closest to its coastline. For these sources, beaching on the shorelines adjoining the Gulf of Aqaba is not observed in June and from August to October. For the remaining months, a measurable impact is observed on the shorelines adjoining the Gulf of Aqaba, from oil particles originating from $S_4$ (January–March and May), $S_{5}-S_{6}$ (February), $S_7$ (February–May, July and October–December) and $S_8$ (February, March and May). A substantial impact on the NEOM shoreline extending from The Line to Sharma is observed from the oil particles originating from $S_{4}$ (November), $S_5$ (October–November) and $S_7$ (October and December). Additionally, beaching of oil on the segment extending from the airport to Duba is not observed for $S_8$ during (January–May, November and December) and for $S_5$ during (January–August). Overall, the results indicate that individual sources near the coastal may have severe impacts away from their location, as measured by the volume fraction of oil beached, and their impact may strongly depend on the seasonal variations of meto-cean conditions.

**Figure 5:**
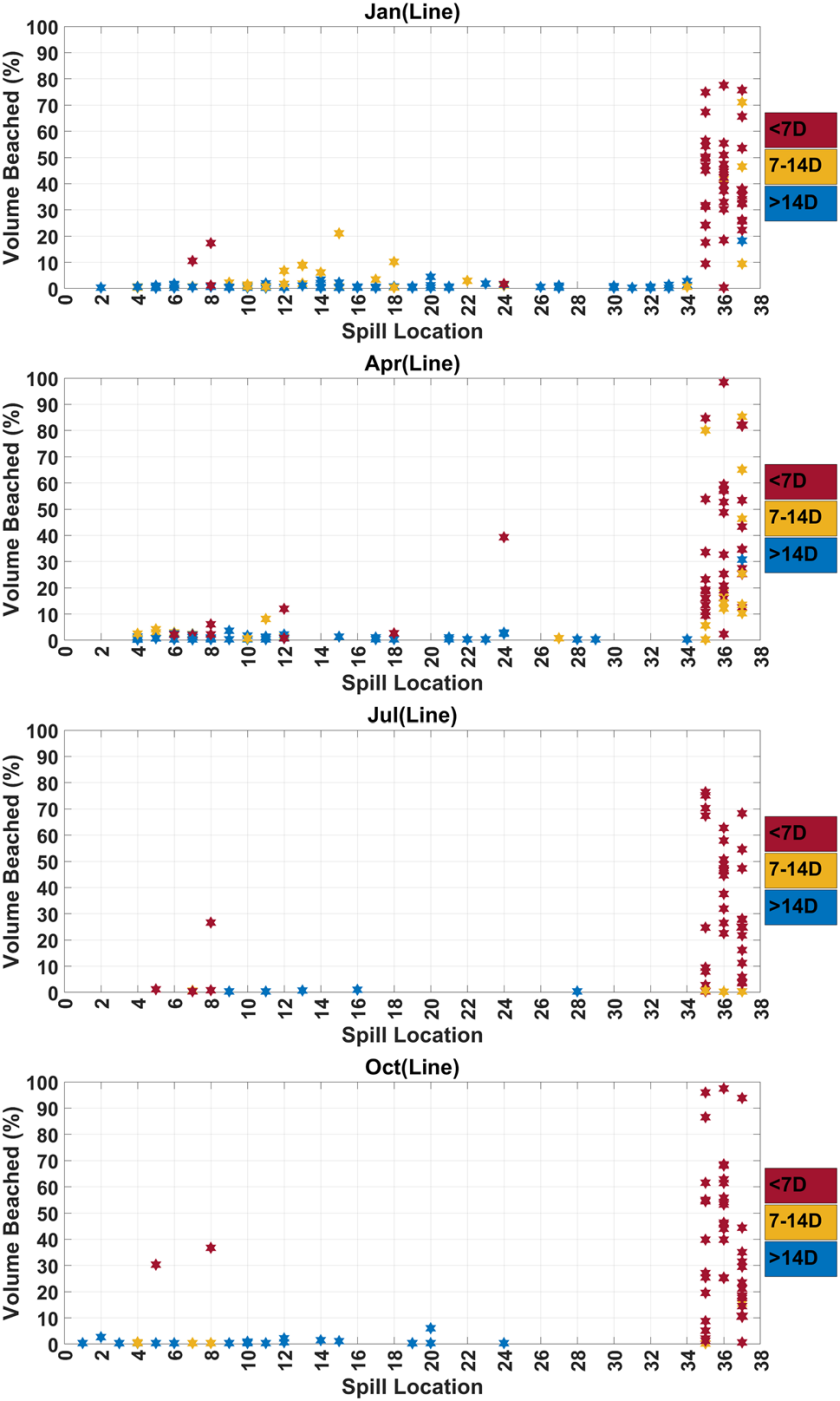
Histograms of the volume fractions beached at the shorelines of The Line. Predictions from all release sources and events are classified (using colors) in terms of the corresponding arrival times. Plots are generated for release events occurring during the January, April, July and October months, as indicated.

### Risk analysis for specific sites

**Figure 6:**
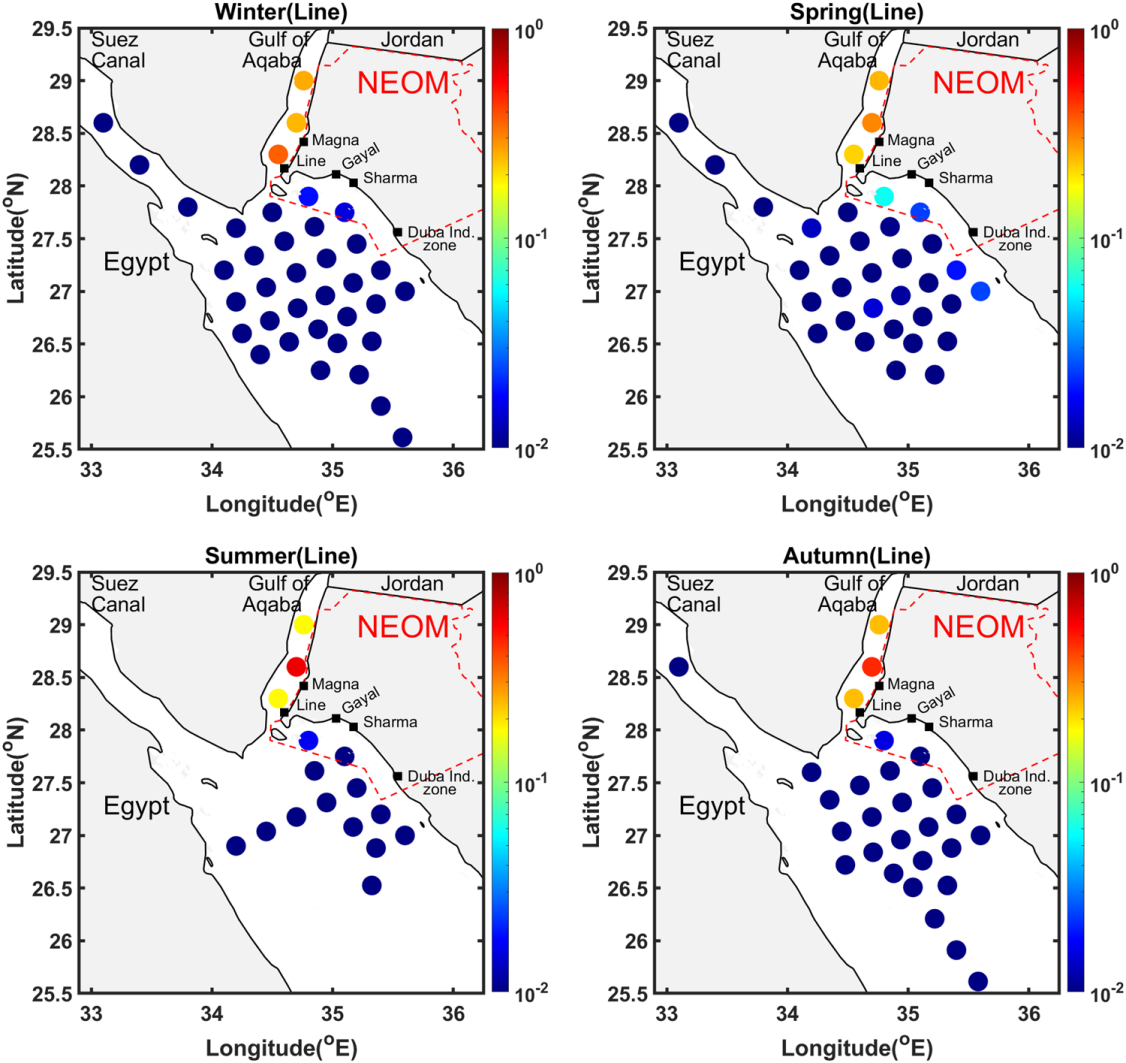
Risk probabilities for shorelines of The Line. The probabilities, estimated using Eq. 1, characterize the region of dependence of the overall risk. Plots are generated for release events occurring during the four seasons, as indicated.

**Figure 7:**
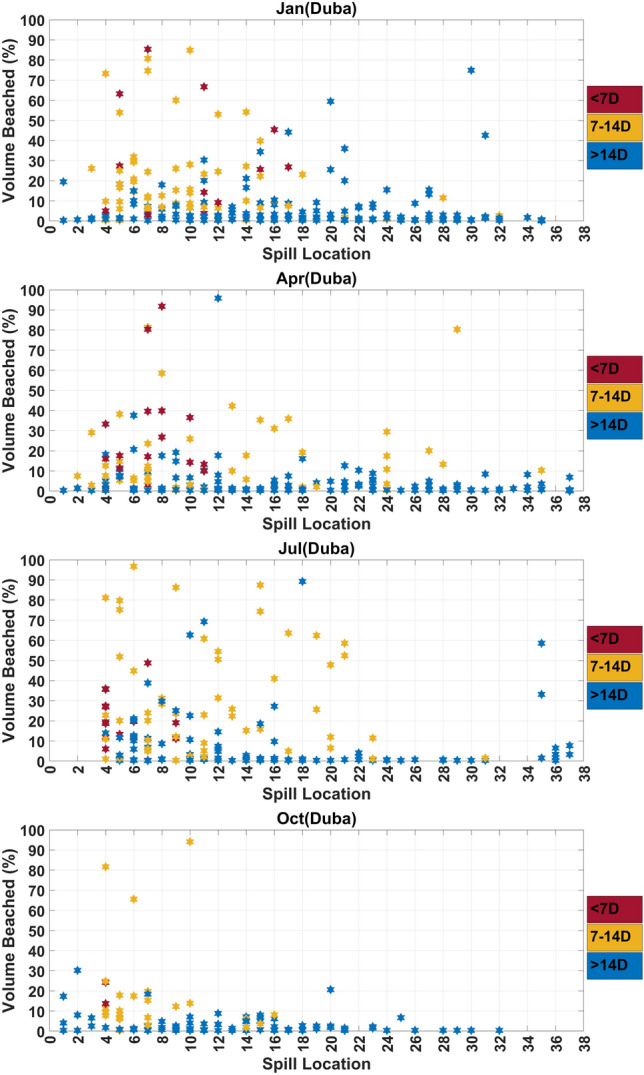
Histograms of the volume fractions beached at the shorelines of Duba. Predictions from all release sources and events are classified (using colors) in terms of the corresponding arrival times. Plots are generated for release events occurring during the January, April, July and October months, as indicated.

**Figure 8:**
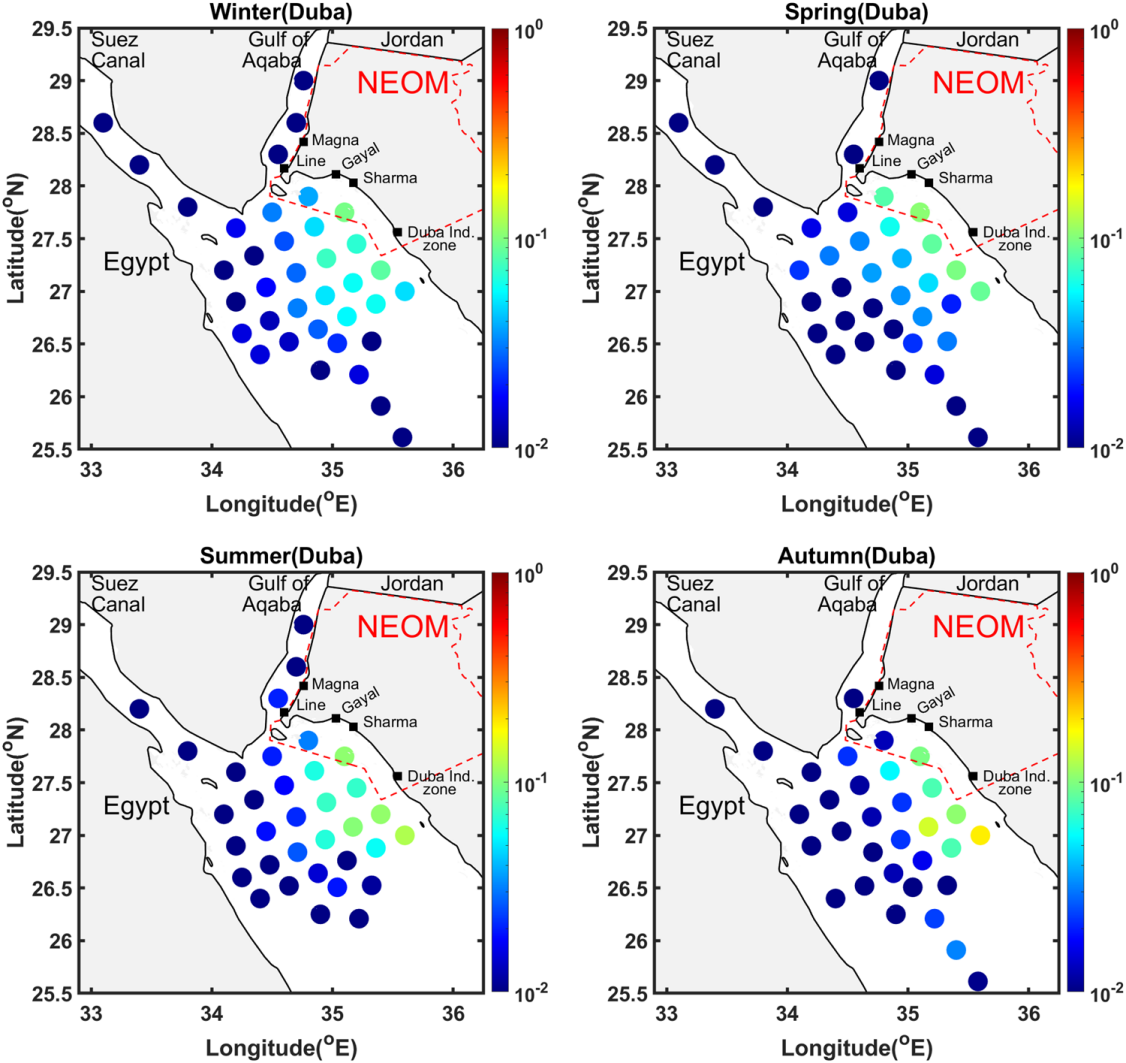
Risk probabilities for shorelines of Duba. The probabilities, estimated using Eq. 1, characterize the region of dependence of the overall risk. Plots are generated for release events occurring during the four seasons, as indicated.

The risk associated with the individual release sources is now analyzed for specific sites along the Neom coast, namely The Line, Duba, Sharma, Gayal and Magna. Figures 5, 7 and Supplementary Figures S5–S9 plot the histograms of volume fractions for each source and release event considered, showing the amount of volume beached and the corresponding arrival time class (classified using colors), during the months from January to December. Figures 6, 8 and Supplementary Figures S10–S12 depict the (inverse) risk probabilities for each of the specific sites considered. These probabilities characterize the region of dependence of the spill risk, as estimated using Eq. 1.

### The Line

Figure 5 and Supplementary Figure S5 plot histograms of the volume fractions beached at the shorelines of The Line, where predictions from all the release sources and events are classified in terms of the corresponding arrival times. The histograms present a uni-modal distribution of the volume fractions with tails varying from approximately 10–80%. The spills originating primarily from sources $S_{35}-S_{37}$ are characterized by the highest severity (low arrival times and high volume fractions) amongst other sources. During the months of April and from September–December, the volume fraction of oil released from source $S_{35}$ and beached around The Line may rise to 85% by the end of the first week. The volume fraction of oil released from source $S_{36}$ and beached around The Line is greater than 60% over the whole year except during April, June and October (greater than 90%). The volume fraction of oil released from source $S_{37}$ is greater than 50% throughout the year, except during the months of June and August (around 20%), by the end of first week. The prevailing northwards currents^4^ towards The Gulf of Aqaba tend to quickly push oil released from $S_8$ towards The Line; in March, the volume fraction may rise to more than 90%. However, the volume fractions remain less than 50% for the whole year except for March, June and September. The segments around The Line may be weakly affected by oil originating from $S_8$ in June and September. Additional events having early arrival times are associated with $S_{18}$ and $S_{24}$, which are located close to the northern tip of The Red Sea between The Gulf of Aqaba and The Gulf of Suez (near Sharm El-Sheikh). Here, the transport of spilled oil towards The Line is promoted by the prevailing coastal currents, which dominate the circulation during the months from December to May. The arrival times fall within 1–2 weeks during these months. Specifically, the arrival time is less than 1 week during December, February, and April for $S_{18}$, and during January and April for $S_{24}$. Events with short arrival time (less than 1 week) are also associated with $S_{5}$ (in March and October) with volume fractions of around 40%. However, very few sources among $S_9-S_{34}$ are characterized by moderate arrival times (2–3 weeks), and generally have low severity in terms of amount of beached oil (volume fractions less than 10%).

Figure 6 depicts the seasonal distribution of risk probabilities, estimated using Eq. 1 for oil beached around The Line. Sources $S_{35}-S_{37}$, located in The Gulf of Aqaba, are responsible for the largest risk. The risk associated with $S_{36}$ is the highest amongst $S_{35}-S_{37}$ in spring, summer and autumn seasons, whereas the risk associated with $S_{35}$ is highest in winter. The risk associated with the remaining sources is appreciably smaller than that observed for $S_{35}-S_{37}$. In addition, the associated probabilities are very small, except possibly for sources $S_5$–$S_8$ for which appreciable values may occur. Overall, the results of Figures 5 and 6 indicate that for The Line, the risk is primarily dominated by sources located in the Gulf of Aqaba, followed to a lower extent by sources located close to its shoreline.

Spills originating from sources located in the Gulf of Aqaba generally lead to severe events, with a large fraction of the oil released beaching within a short period (< 7 days) from the time of the release. Consistent with the histograms in Fig. 5, sources located in the Red Sea and close to the NEOM shoreline may result in severe impact on The Line, but these events have low probability of occurrence, leading to small risk values reported in Fig. 6.

### *Duba*

Figure 7 and Supplementary Figure S6 plot histograms of the volume fractions beached at the shorelines of Duba. In contrast to those corresponding to The Line, the results indicate that the shoreline surrounding Duba is vulnerable to sources located in the entire region facing its coast. This is reflected by the fact that multiple events with severe impacts are observed for sources $S_{4}-S_{21}$, which are located in the open waters facing the NEOM coast. As expected, sources $S_{4}-S_{8}$, which lie closest to the NEOM coastline are characterized by higher impacts and shorter arrival times than $S_{9}-S_{21}$. Overall, sources $S_{4}-S_{21}$ lead to events of various severity, and the histogram accordingly exhibits a large scatter over the corresponding segment. The Duba region appears to be less susceptible to sources lying in the Gulf of Suez, which are far away from the Duba region, and in the Gulf of Aqaba, except for $S_{35}$ located at the tip of the Gulf which may result with low probability in a large fraction of oil beached near Duba.

Figure 8 illustrates the seasonal distribution of the aggregate probability of volume beached corresponding to oil spills that affect the Duba shoreline. As opposed to the Line’s shoreline, which is primarily affected by the release sources in The Gulf of Aqaba, sources $S_{4}-S_{12}$ and $S_{14}-S_{15}$ are characterized by the highest aggregate probabilities of volume beached at the Duba shoreline, throughout the year. The aggregate probability of $S_{4}$ is the highest in autumn season. Few of the sources located in The Gulf of Aqaba are characterized by insignificant probabilities (< 0.01) in the spring ($S_{36}$) and autumn ($S_{36}-S_{37}$) seasons. The majority of the sources ($S_{26}-S_{34}$) located farther from the Saudi coastline and closer to Egyptian coast or in the Gulf of Suez are characterized by the lowest probabilities throughout the year.

### Magna, Sharma and Gayal

For Magna, Sharma and Gayal, histograms of the volume fractions of oil beached and of risk distributions are shown in Supplementary Figures S7–S8 (Magna), S9–S10 (Sharma) and S11–S12 (Gayal). For the sake of brevity, the main takeway findings are provided in this section.

The plots for Magna indicate similarities to those obtained for The Line, where Magna’s shoreline is seen to be predominantly at risk from the release sources in the Gulf of Aqaba. These sources tend to be associated with the highest impact, with short arrival times and large volumes of oil beached. Furthermore, the results corresponding to Sharma and Gayal exhibit key similarities with those obtained for Duba. Specifically, the Sharma and Gayal shorelines are primarily vulnerable to the release sources nearest to the Saudi coast, with decreasing risk from the release sources located far from the Saudi coastline. The Gayal shoreline is generally protected from oil spills, which may be attributed to the nearby islands and the shape of its bay. In contrast, Sharma’s coastline is more exposed to oil spills because its geographic location features an open bay. Therefore, more moderate and high severity events are reported for Sharma, from the release sources lying in the first two rows facing the NEOM shoreline.

## Conclusion

We conducted a risk assessment associated with accidental oil spills from fixed sources on the NEOM shoreline, focusing in particular on key sites and installations. For each potential release site, oil spill simulations were conducted over a 28-day period, starting from the first, tenth and twentieth days of each month, over five consecutive years ranging from 2013 to 2017. The simulations were carried out using the MOHID’s oil spill model, driven with validated high-resolution met-ocean fields of the Red Sea. The risk associated with each release event was characterized by the minimum time for an oil particle to reach the coast, and by the percentage of the total volume of oil released that was beached on the NEOM coast at the end of the simulation period.

The results indicate that spills originating in the Gulf of Aqaba are characterized by short arrival times and high volume fractions, making them the most hazardous to the NEOM shoreline. This occurs throughout the year except for the summer months, when the prevailing southwards currents in the Gulf of Aqaba tend to push the oil slicks towards the Tiran and Sanfir islands, which does not minimize their potential impact because these islands are key sites for tourism. Release sources located in the open water closest to the Saudi Arabian shoreline are generally associated with short arrival times, except during the months of September and October. These release sources impact NEOM’s islands and the region connecting Sharma to Duba throughout the year. On the other hand, these release sources have weak impact on the NEOM shoreline lying in the Gulf of Aqaba, between June and December. Release sources located in the Gulf of Suez have a slight impact on the NEOM shoreline during the months of January, Februrary and March. Finally, spills originating from release sources located in the open waters close to the Egyptian coast are characterised by moderate arrival times and low volume fractions, throughout the year.

The shorelines of Magna and The Line are subject to a similar response to the oil spill scenarios considered, where both were vulnerable to the release sources located in the Gulf of Aqaba. Moreover, release events south of Tiran and near Sanafir islands may have a significant impact on The Line’s shore, particularly during winter and more so in spring. Duba, Sharma and Gayal’s shorelines exhibit similar behavior in response to accidental oil spills from the sources considered. Specifically, release sources lying closest to the Saudi Arabian shoreline have the biggest impact on the shorelines of these sites. The releases are characterized by short arrival times and large fractions of volume beached. The adjacent release sources also exhibit a considerable impact, that is weaker during the Autumn months. These release events are typically associated with medium severity arrival times and fractions of volume beached. Finally, Duba, Sharma and Gayal’s shorelines appear to be at low risk from accidental oil spill scenarios originating from release sources near the African shoreline during the summer and autumn seasons.

### Supplementary Information

Supplementary Figures.

## Data Availability

The datasets used and/or analyzed during the current study are available from the corresponding author upon reasonable request.
