# Supplementary material for: Oil spill risk analysis for the NEOM shoreline
Source: Sci Rep. 2024 Mar 19;14:6623. doi: 10.1038/s41598-024-57048-4 (PMC10951341; doi:10.1038/s41598-024-57048-4)
Supplement: Supplementary file 1 — Supplementary Figures. [file 41598_2024_57048_MOESM1_ESM.pdf]

Supplementary Figures

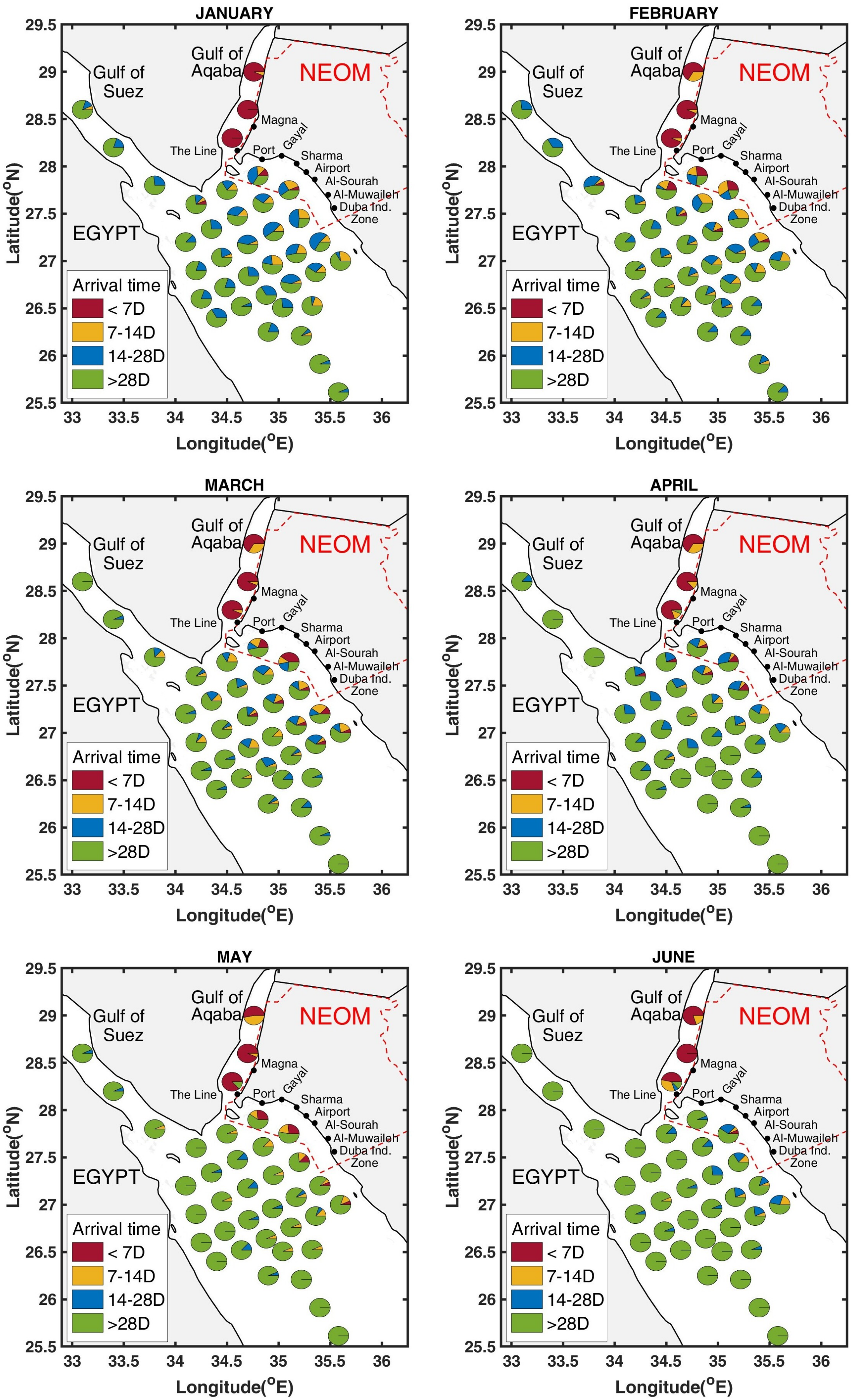
JANUARY
FEBRUARY
MARCH
APRIL
MAY
JUNE
Gulf of Aqaba
Gulf of Suez
NEOM
EGYPT
Magna
Gayal
The Line
Port
Sharma
Airport
Al-Sourah
Al-Muwaileh
Duba Ind. Zone
Arrival time
< 7D
7-14D
14-28D
>28D
Latitude(°N)
Longitude(°E)
29.5
29
28.5
28
27.5
27
26.5
26
25.5
33
33.5
34
34.5
35
35.5
36

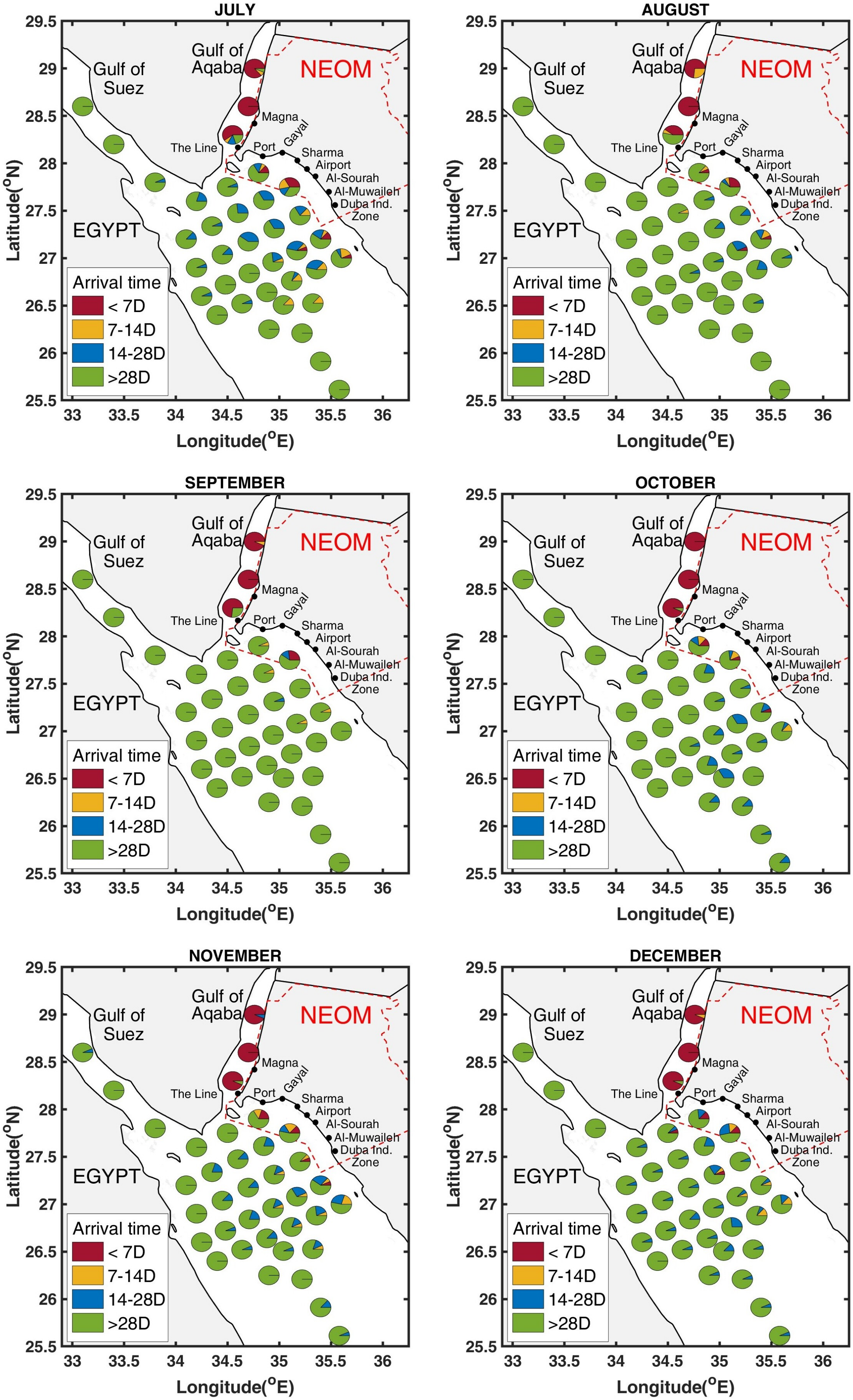

Supplementary Figure 1. Pie charts centered at each release source, representing the corresponding arrival times. The plot title indicates the month in which the releases occur.

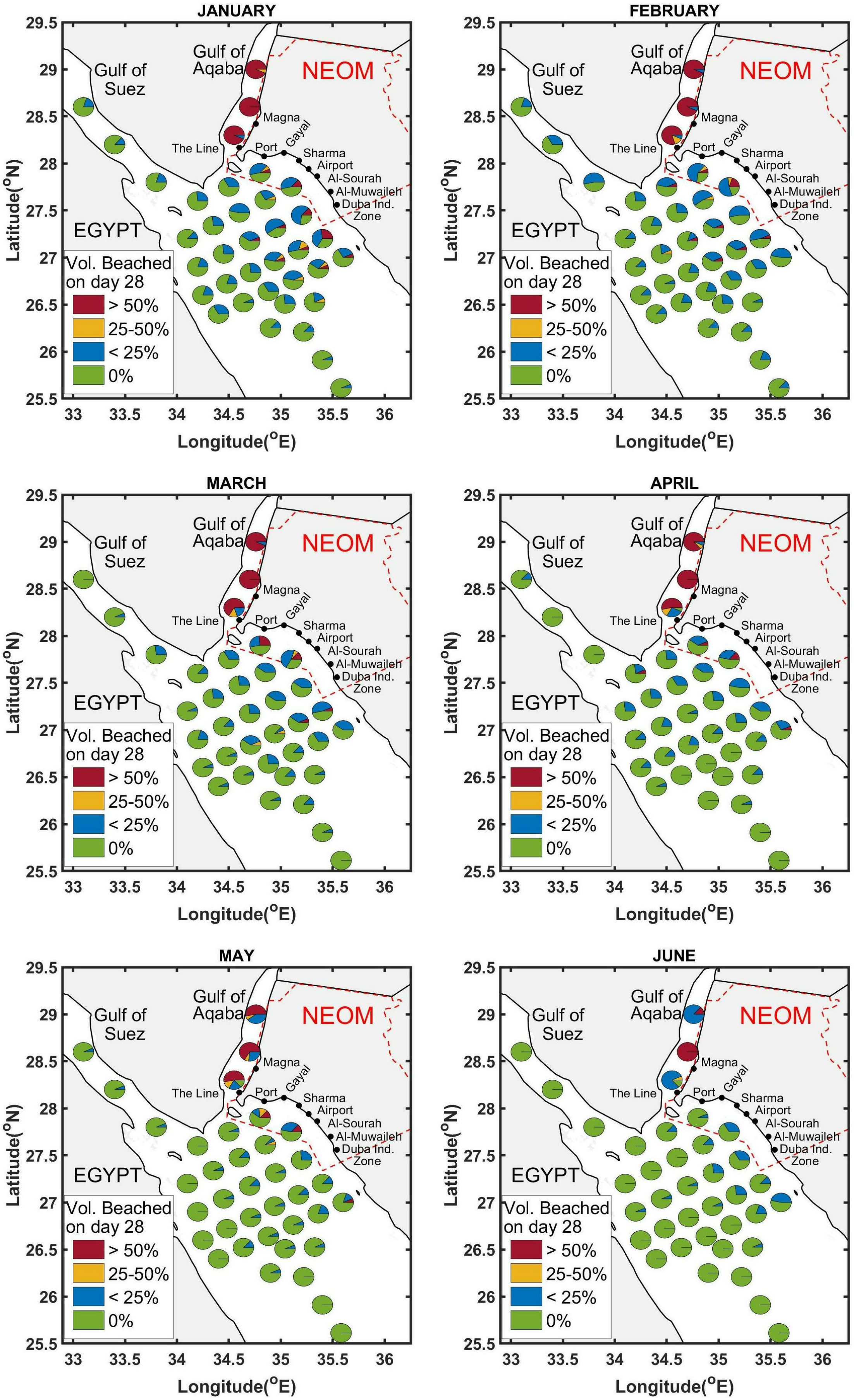
JANUARY
FEBRUARY
MARCH
APRIL
MAY
JUNE
Gulf of Aqaba
Gulf of Suez
NEOM
EGYPT
Magna
Gayal
Port
The Line
Sharma
Airport
Al-Sourah
Al-Muwaileh
Duba Ind. Zone
Vol. Beached on day 28
> 50%
25-50%
< 25%
0%
Latitude(°N)
Longitude(°E)
29.5
29
28.5
28
27.5
27
26.5
26
25.5
33
33.5
34
34.5
35
35.5
36

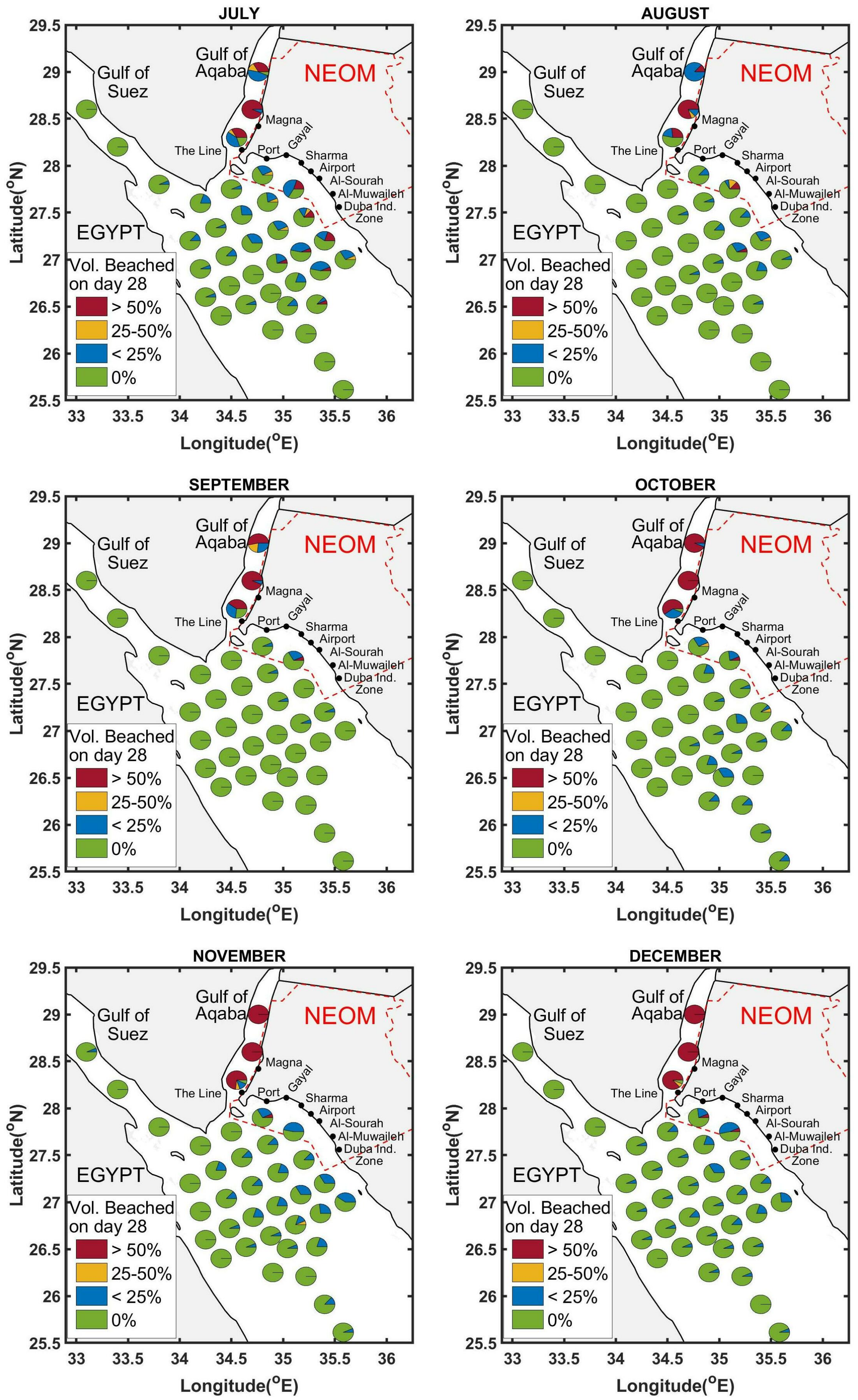

Supplementary Figure 2. Pie charts centered at each release source, representing the corresponding the volume fractions of beached oil particles. The plot title indicates the month in which the releases occur.

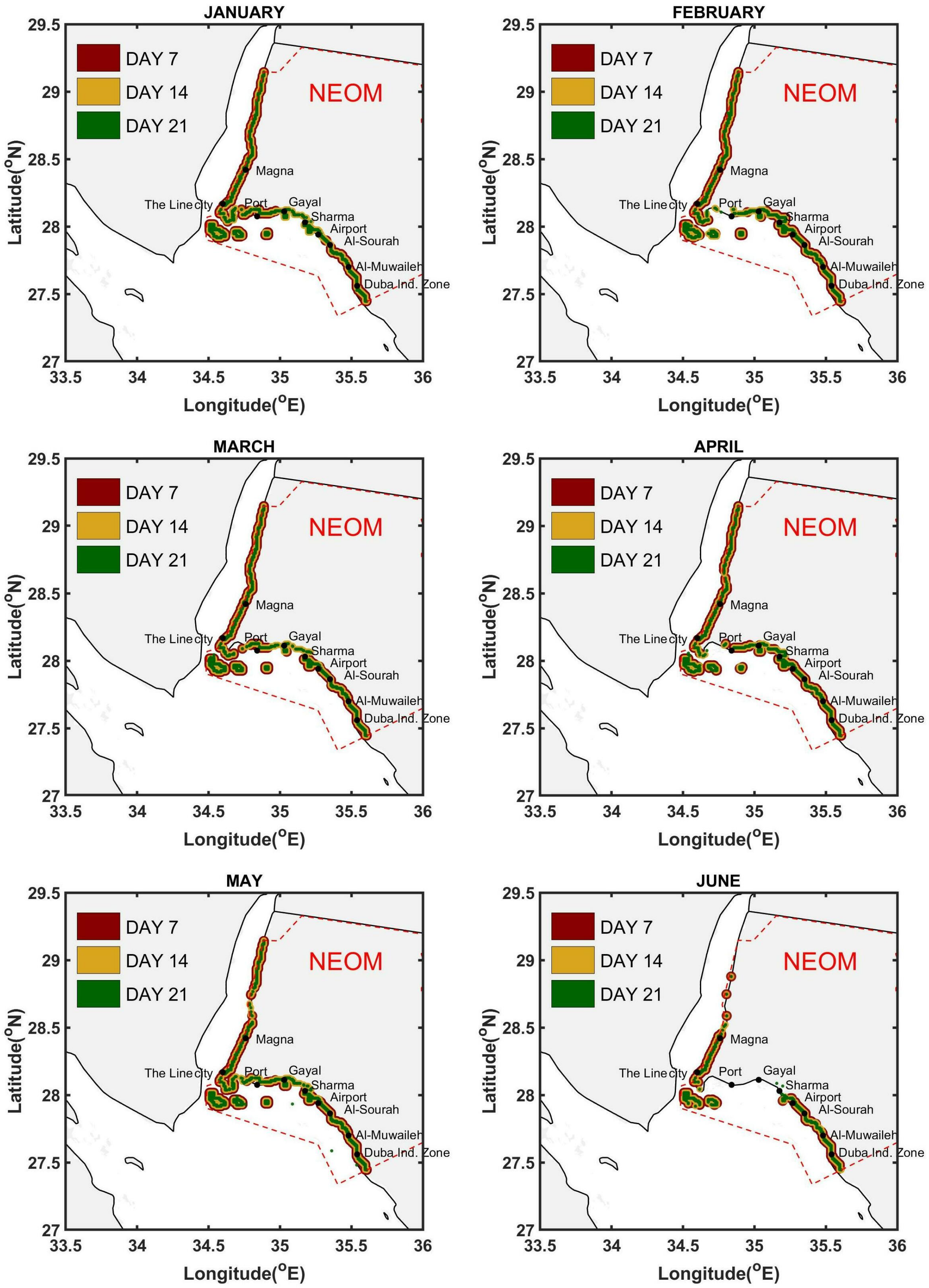
JANUARY
FEBRUARY
MARCH
APRIL
MAY
JUNE
DAY 7
DAY 14
DAY 21
NEOM
Magna
The Line city
Port
Gayal
Sharma
Airport
Al-Sourah
Al-Muwaileh
Duba Ind. Zone
Latitude(°N)
Longitude(°E)
29.5
29
28.5
28
27.5
27
33.5
34
34.5
35
35.5
36

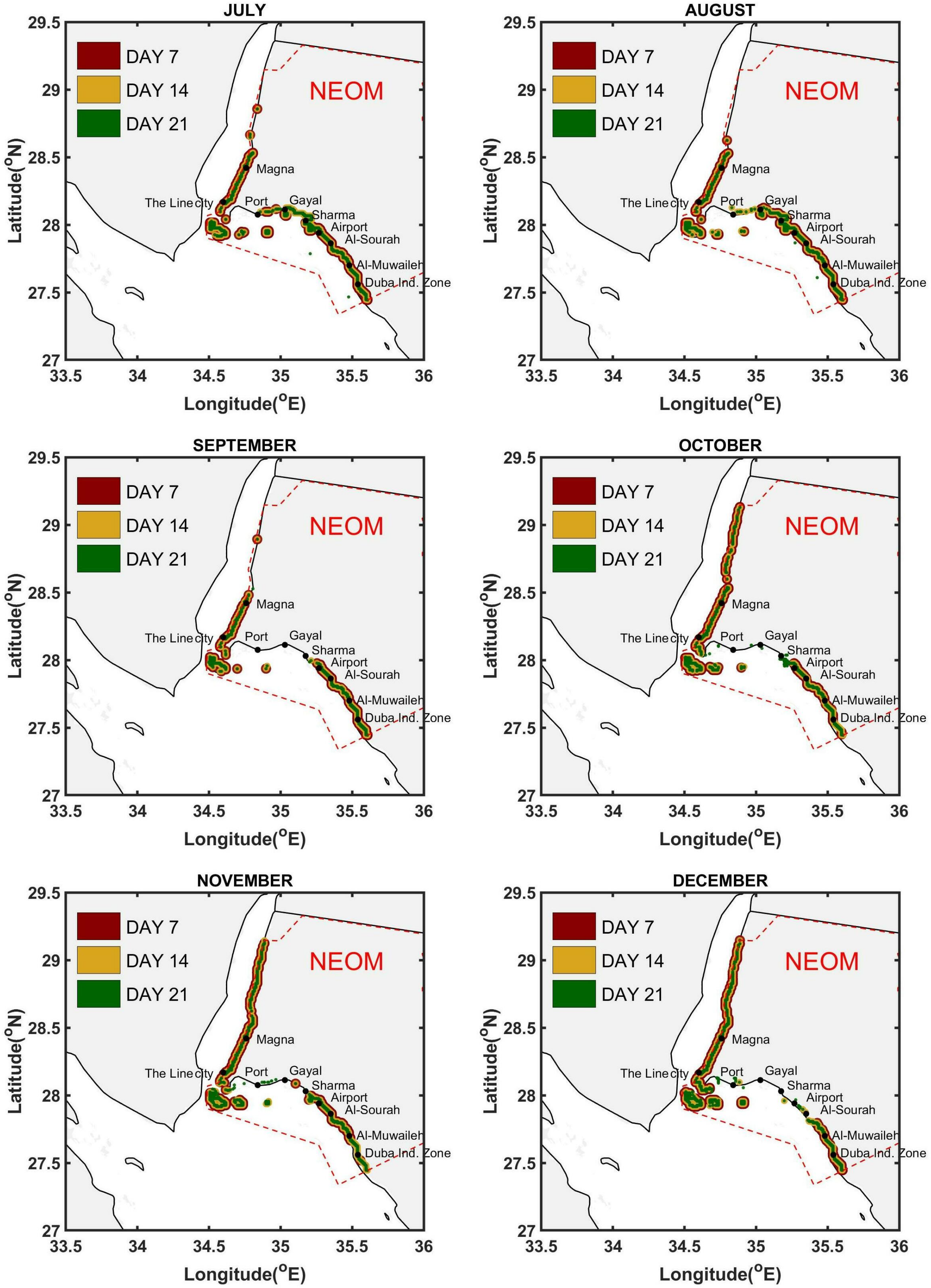

Supplementary Figure 3. Regions of the NEOM shoreline affected by beaching, for 7, 14 and 21 days after the onset of the spill. Particles originating from all release sources are used to generate the contours. The plot title indicates the month in which the releases occur.

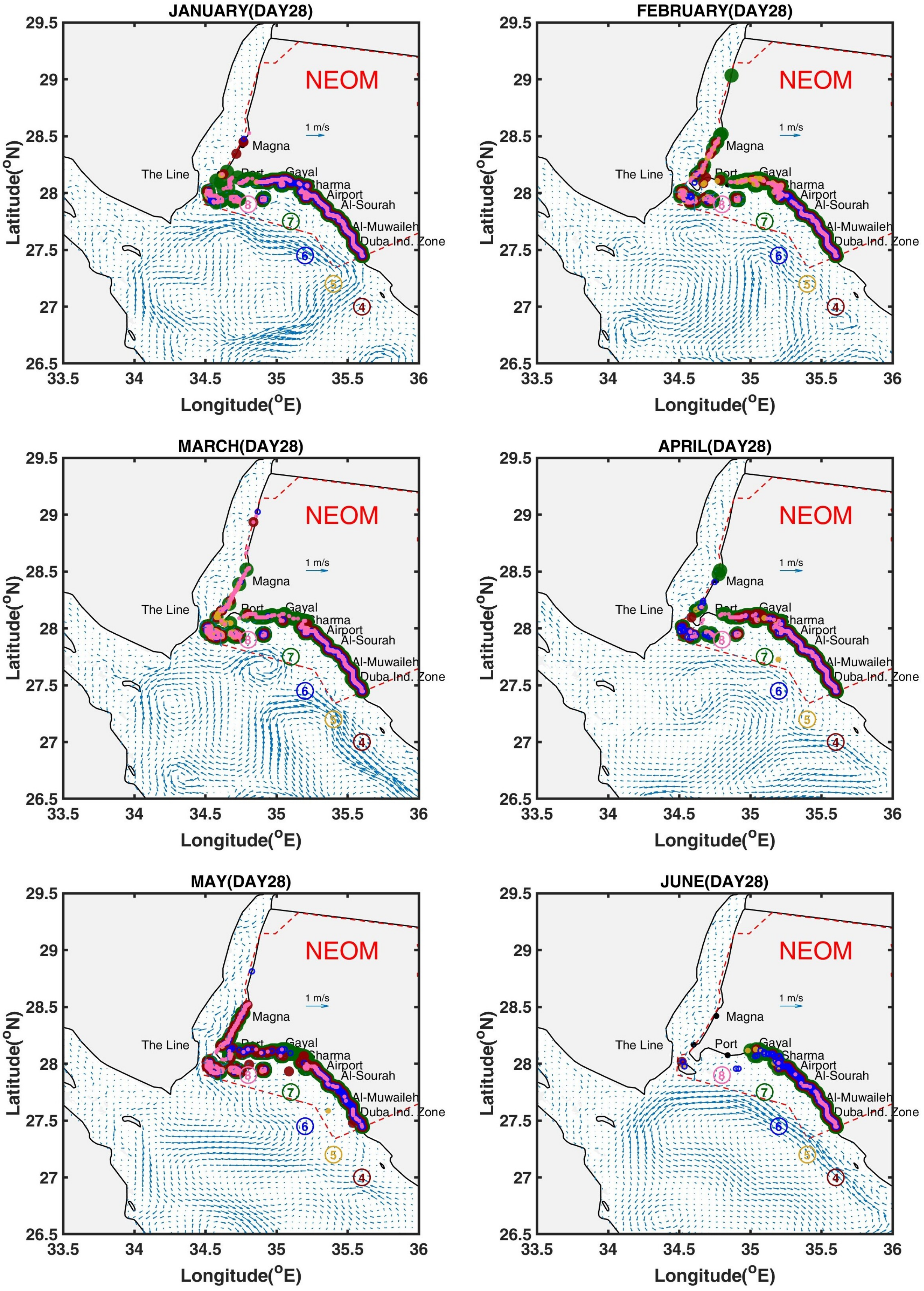
JANUARY(DAY28)
FEBRUARY(DAY28)
MARCH(DAY28)
APRIL(DAY28)
MAY(DAY28)
JUNE(DAY28)
NEOM
1 m/s
Magna
The Line
Port
Gayal
Sharma
Airport
Al-Sourah
Al-Muwaileh
Duba Ind. Zone
Latitude(°N)
Longitude(°E)
29.5
29
28.5
28
27.5
27
26.5
33.5
34
34.5
35
35.5
36

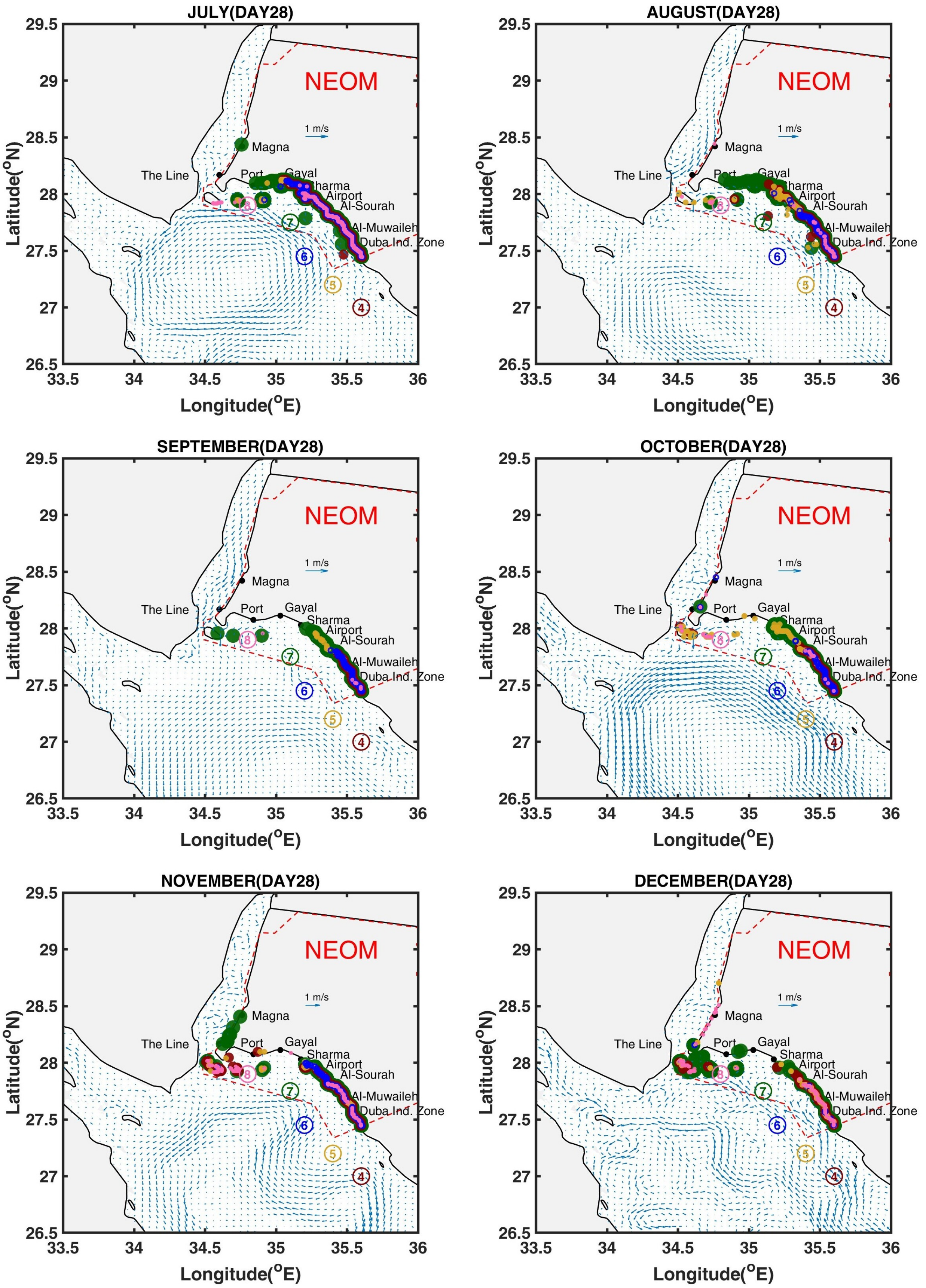

Supplementary Figure 4. Regions of the NEOM shoreline affected by beaching at the end of the 28-day simulation period. The contributions of selected sources are isolated using different color scheme for the individual sources, as indicated. The plot title indicates the month in which the releases occur.

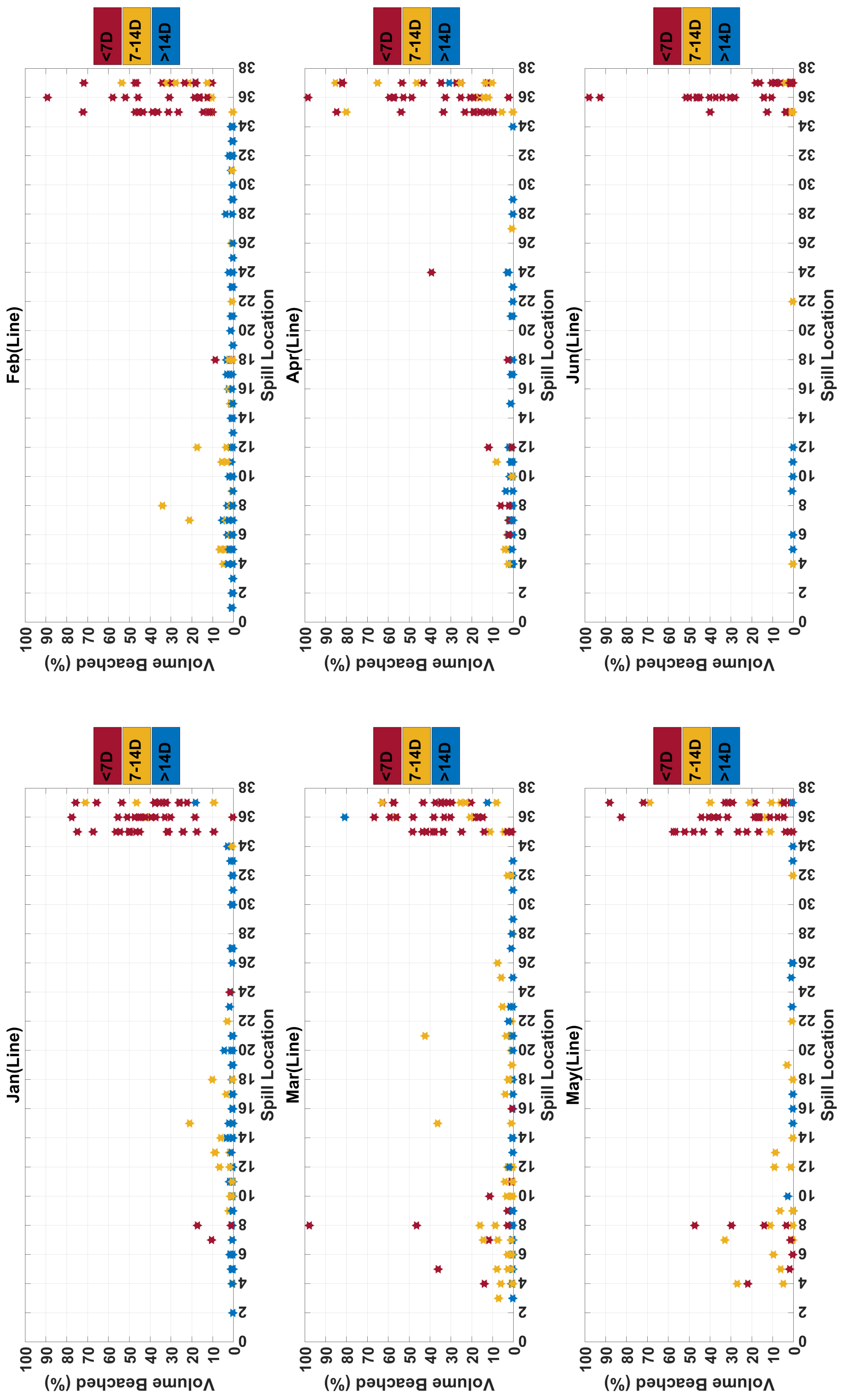
Jan(Line)
Feb(Line)
Mar(Line)
Apr(Line)
May(Line)
Jun(Line)
Volume Beached (%)
Spill Location
<7D
7-14D
>14D

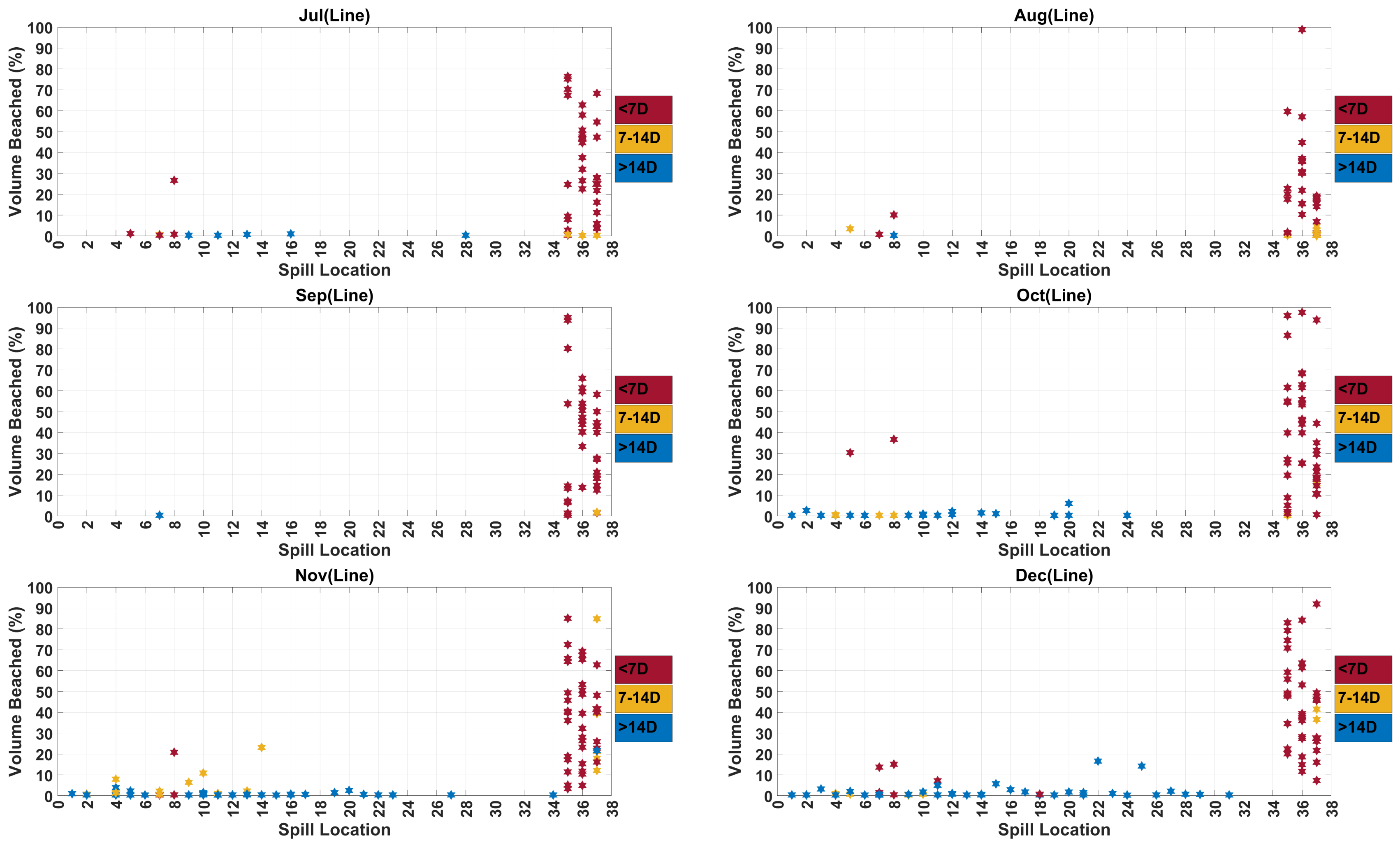

Supplementary Figure 5. Histograms of the volume fractions beached at the shorelines of The Line. Predictions from all release sources and events are classified (using colors) in terms of the corresponding arrival times. The plot title indicates the month in which the releases occur.

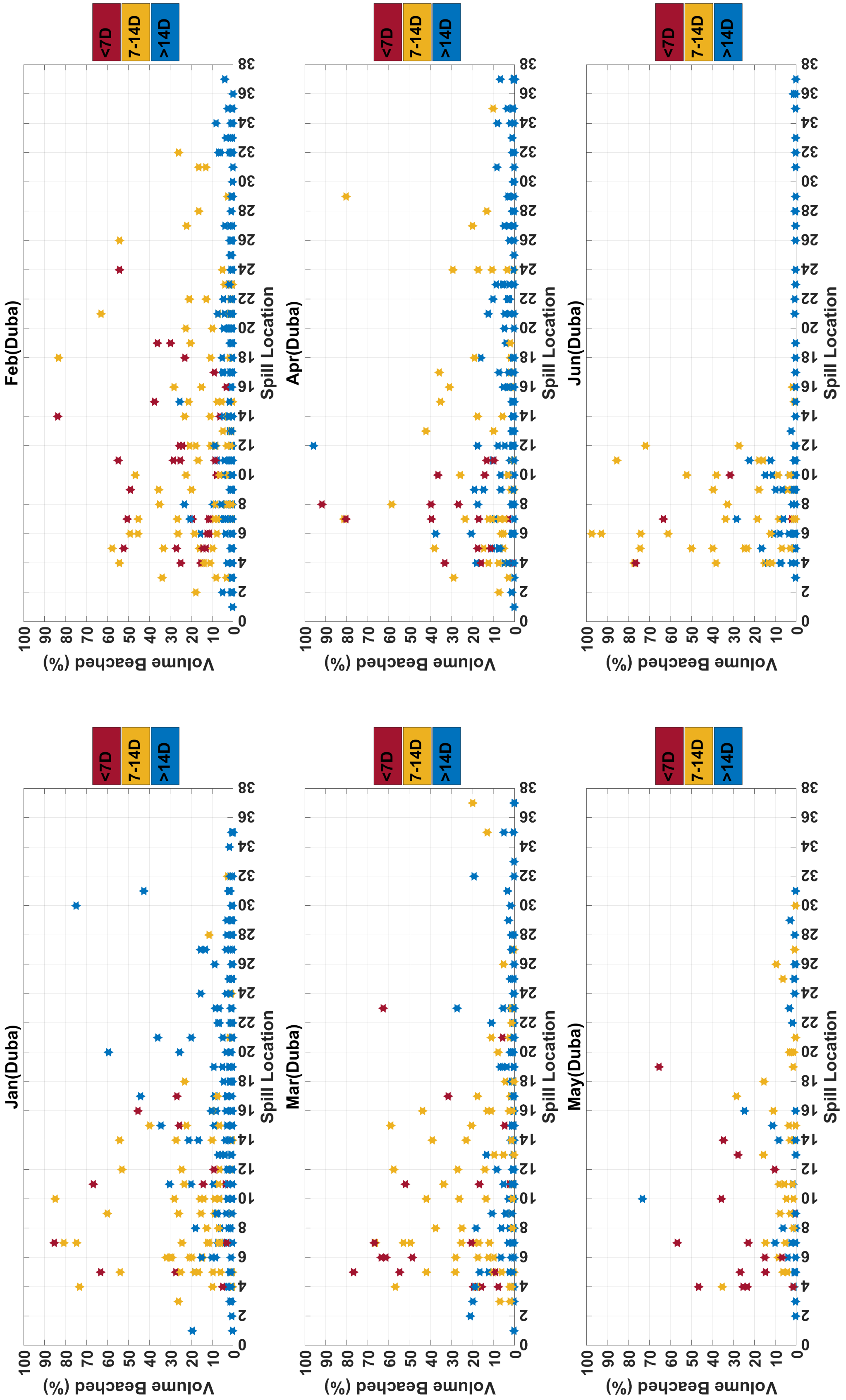
Jan(Duba)
Feb(Duba)
Mar(Duba)
Apr(Duba)
May(Duba)
Jun(Duba)
Volume Beached (%)
Spill Location
<7D
7-14D
>14D

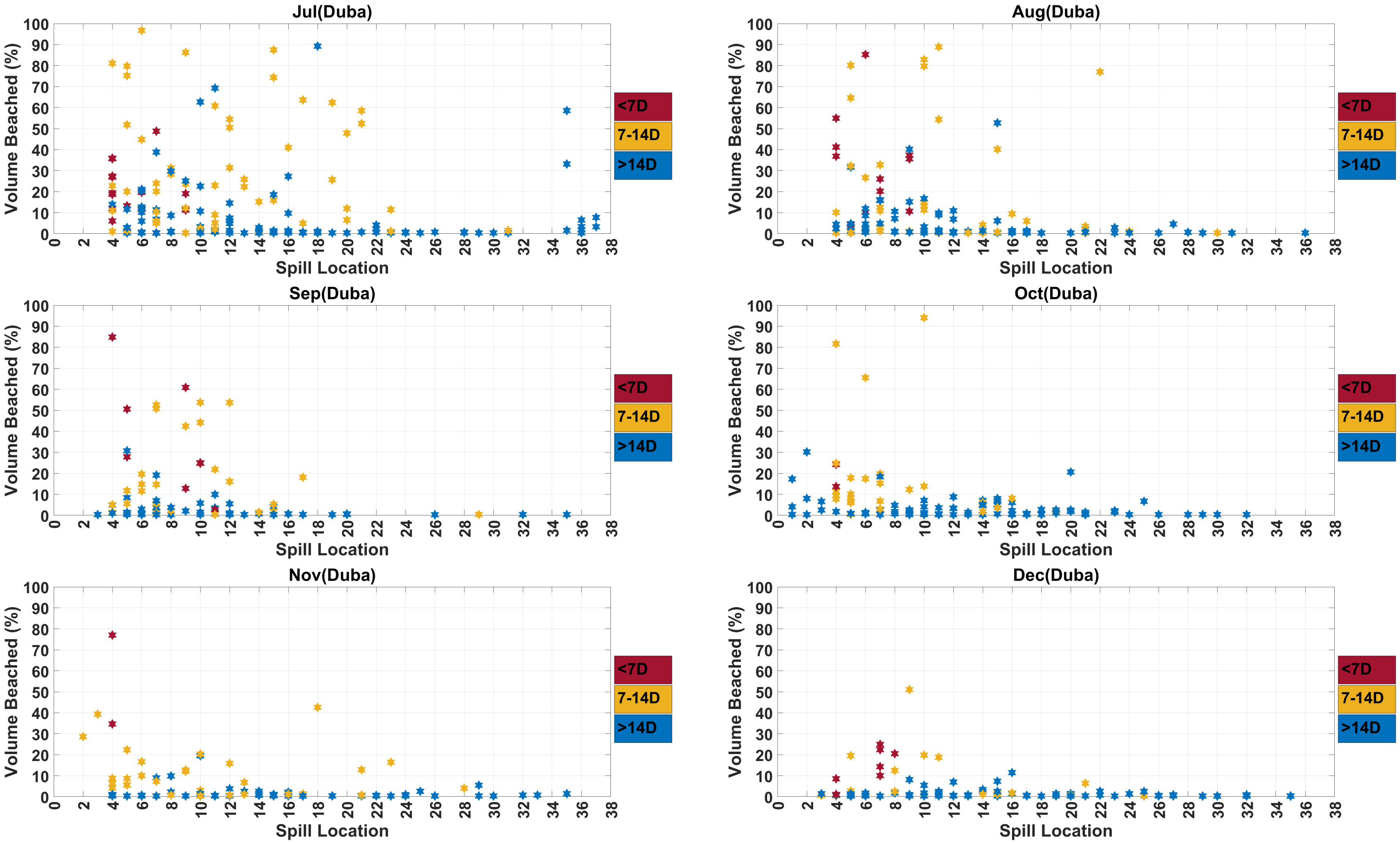

Supplementary Figure 6. Histograms of the volume fractions beached at the shorelines of Duba. Predictions from all release sources and events are classified (using colors) in terms of the corresponding arrival times. The plot title indicates the month in which the releases occur.

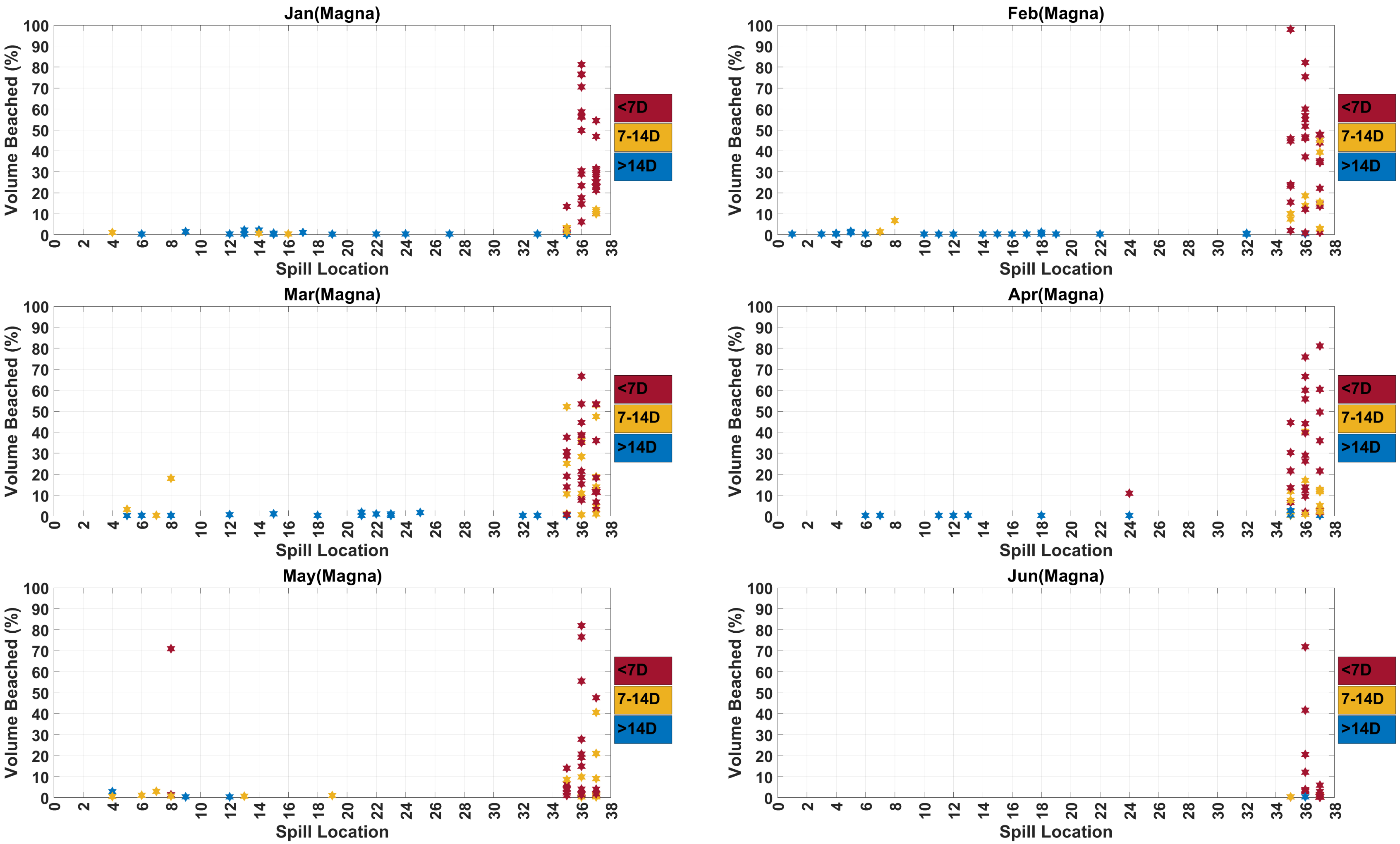

Jan(Magna)
Feb(Magna)
Mar(Magna)
Apr(Magna)
May(Magna)
Jun(Magna)
Volume Beached (%)
Spill Location
<7D
7-14D
>14D

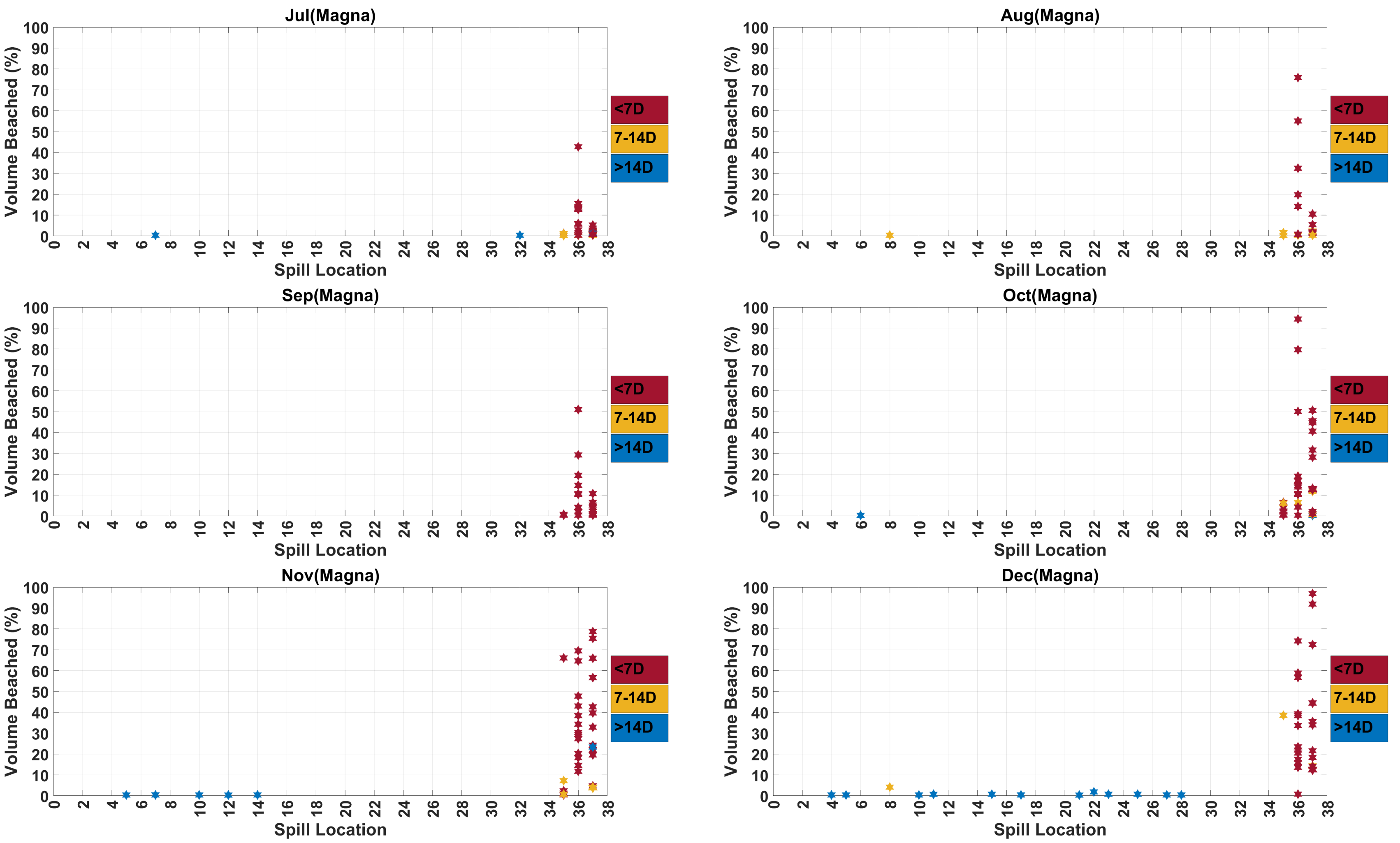

Supplementary Figure 7. Histograms of the volume fractions beached at the shorelines of Magna. Predictions from all release sources and events are classified (using colors) in terms of the corresponding arrival times. The plot title indicates the month in which the releases occur.

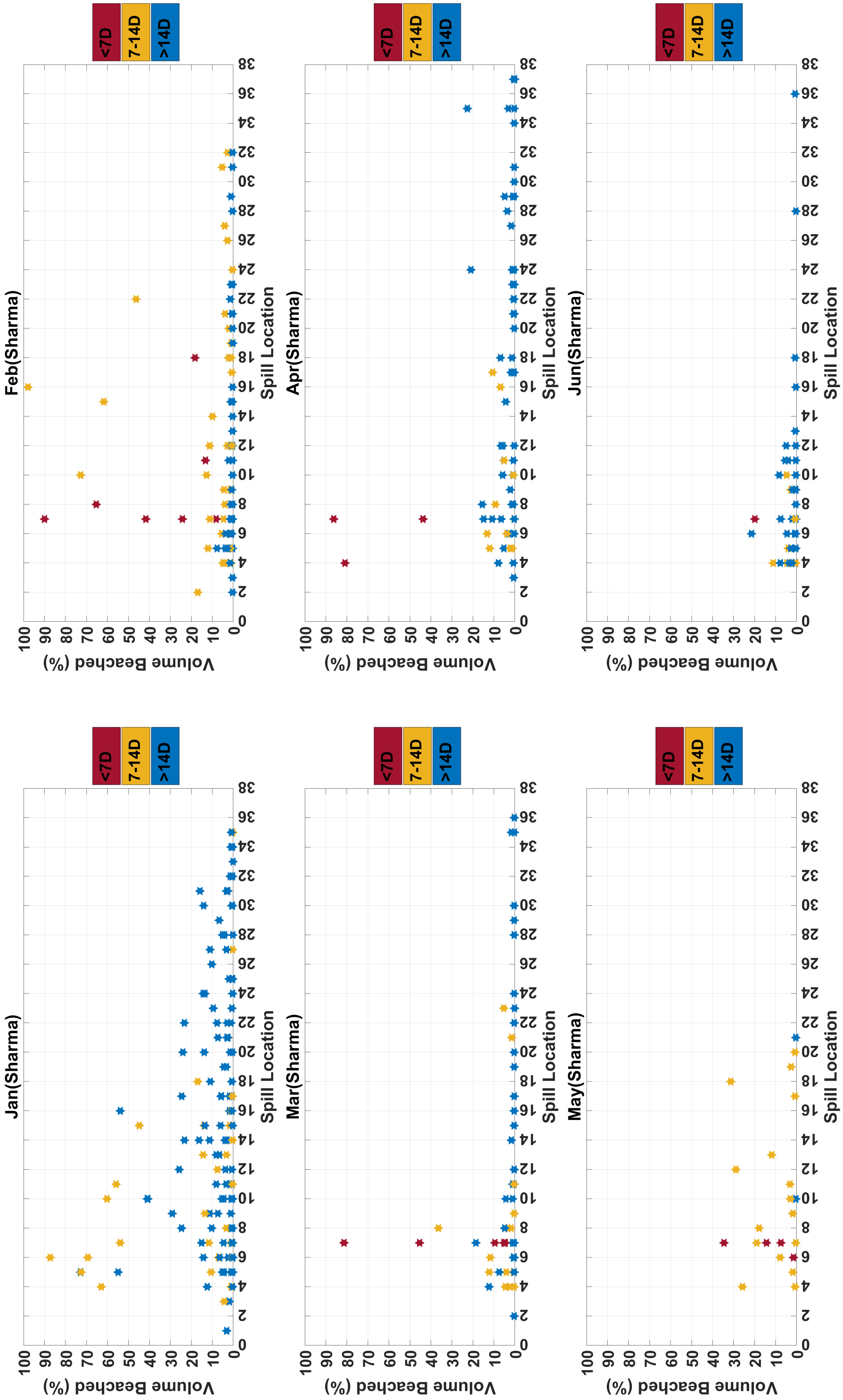
Jan(Sharma)
Feb(Sharma)
Mar(Sharma)
Apr(Sharma)
May(Sharma)
Jun(Sharma)
Volume Beached (%)
Spill Location
<7D
7-14D
>14D

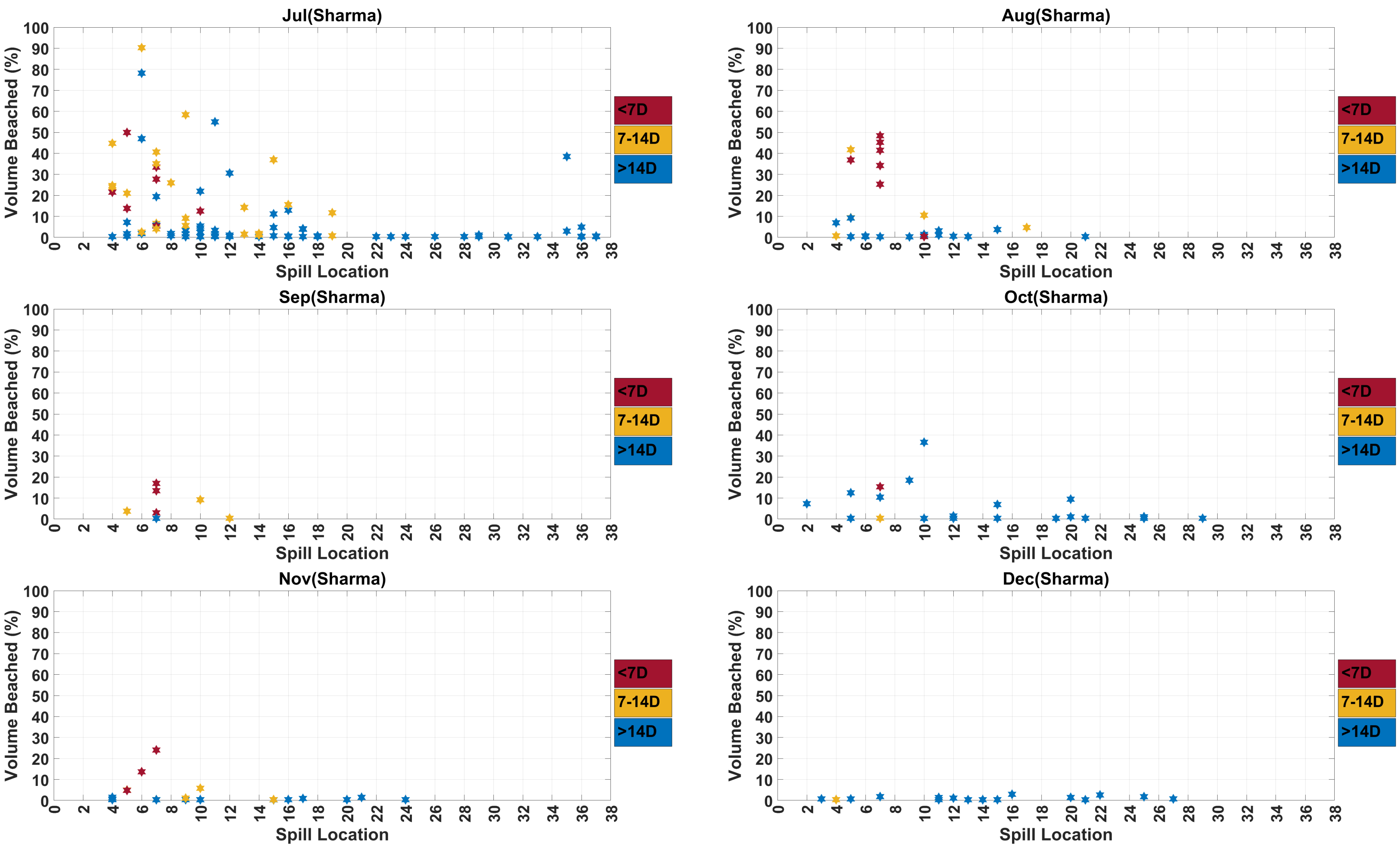

Supplementary Figure 8. Histograms of the volume fractions beached at the shorelines of Sharma. Predictions from all release sources and events are classified (using colors) in terms of the corresponding arrival times. The plot title indicates the month in which the releases occur.

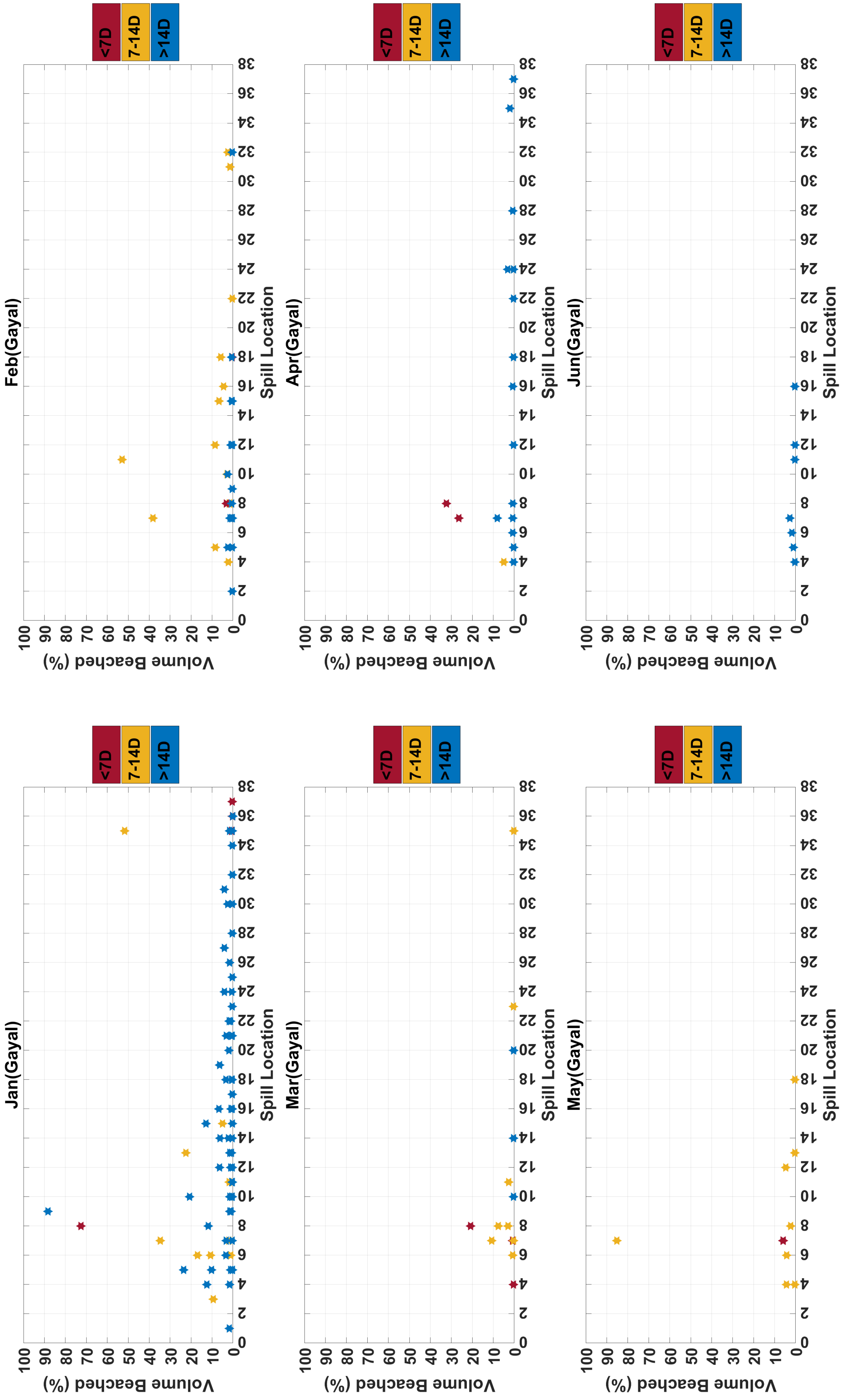
Jan(Gayal)
Feb(Gayal)
Mar(Gayal)
Apr(Gayal)
May(Gayal)
Jun(Gayal)
Volume Beached (%)
Spill Location
<7D
7-14D
>14D

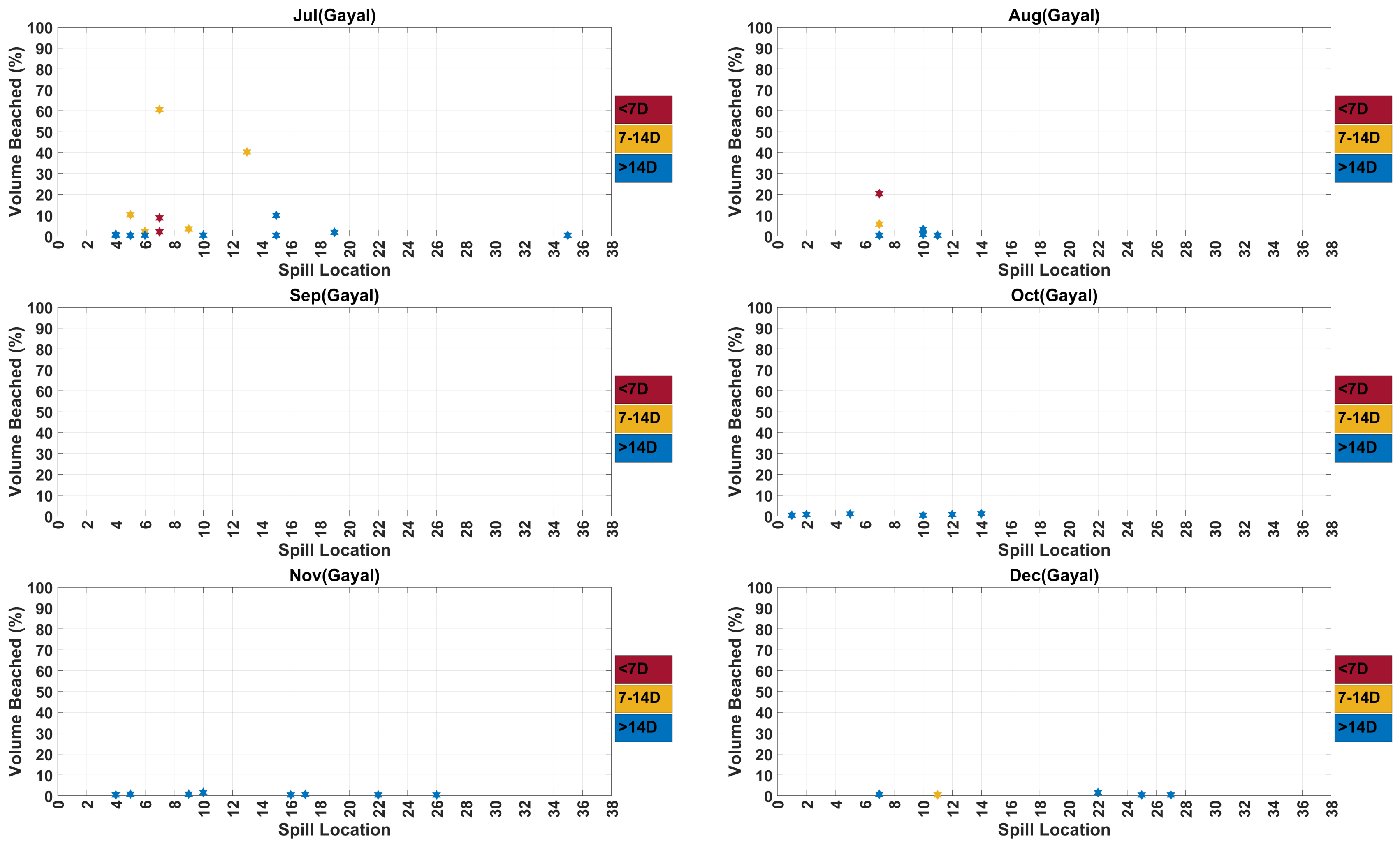

Supplementary Figure 9. Histograms of the volume fractions beached at the shore of Gayal. Predictions from all release sources and events are classified (using colors) in terms of the corresponding arrival times. The plot title indicates the month in which the releases occur.

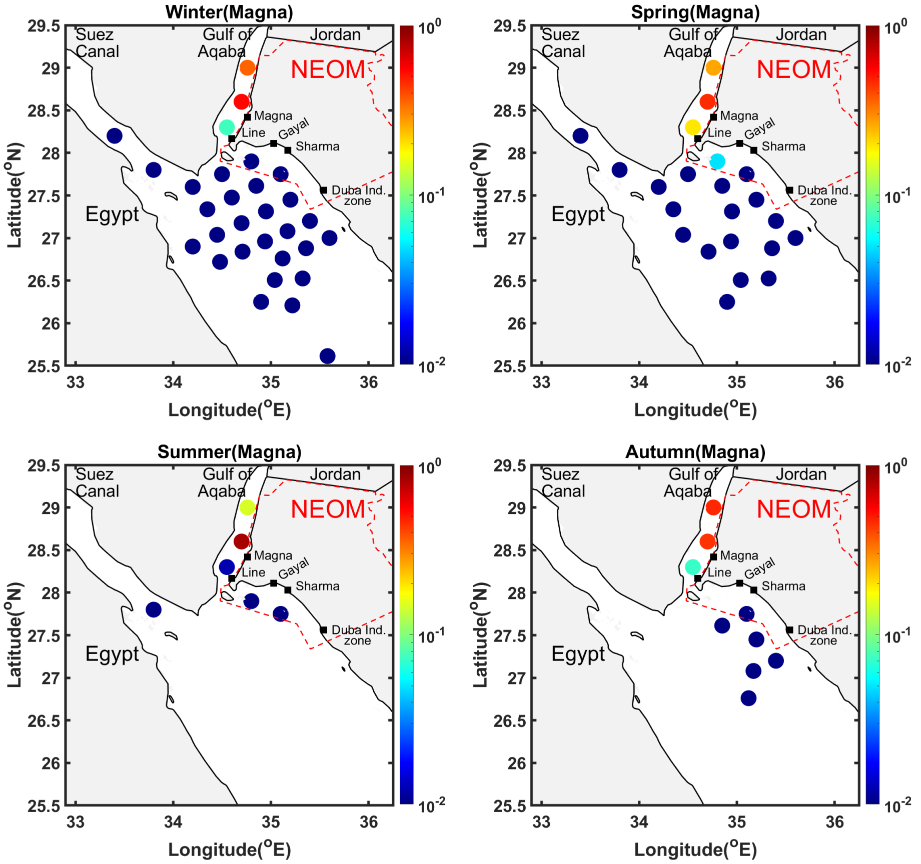

Supplementary Figure 10. Risk probabilities for shorelines of Magna. The probabilities, estimated using Eq. 1, characterize the region of dependence of the overall risk. The plot title indicates the season in which the releases occur.

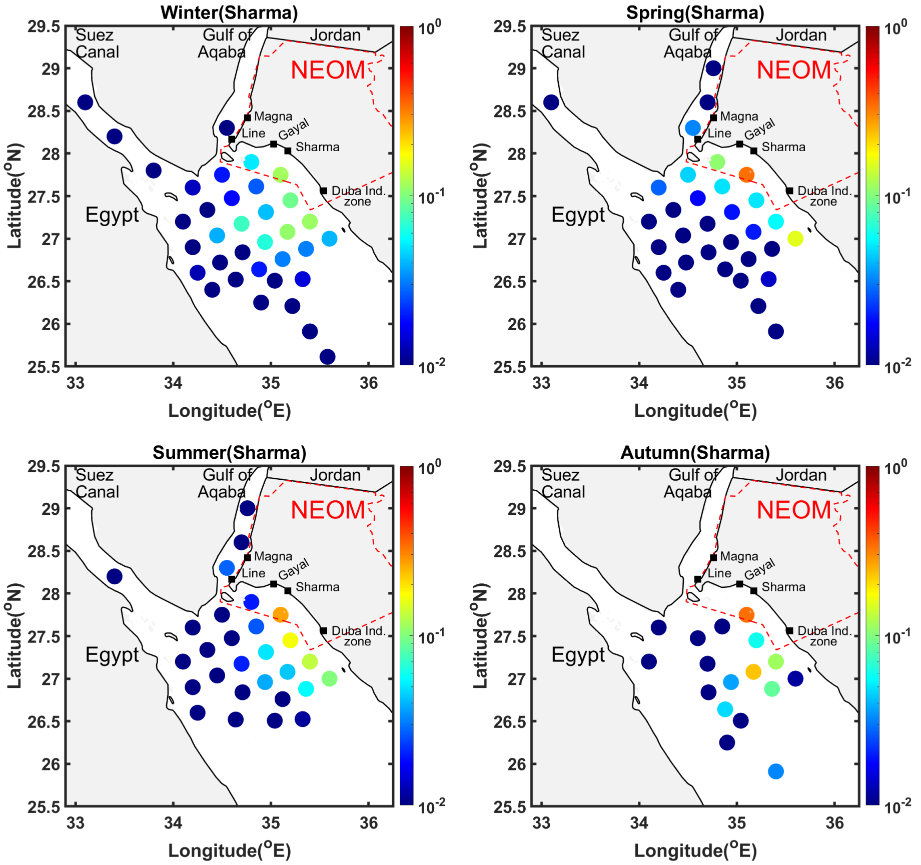

Supplementary Figure 11. Risk probabilities for shorelines of Sharma. The probabilities, estimated using Eq. 1, characterize the region of dependence of the overall risk. The plot title indicates the season in which the releases occur.

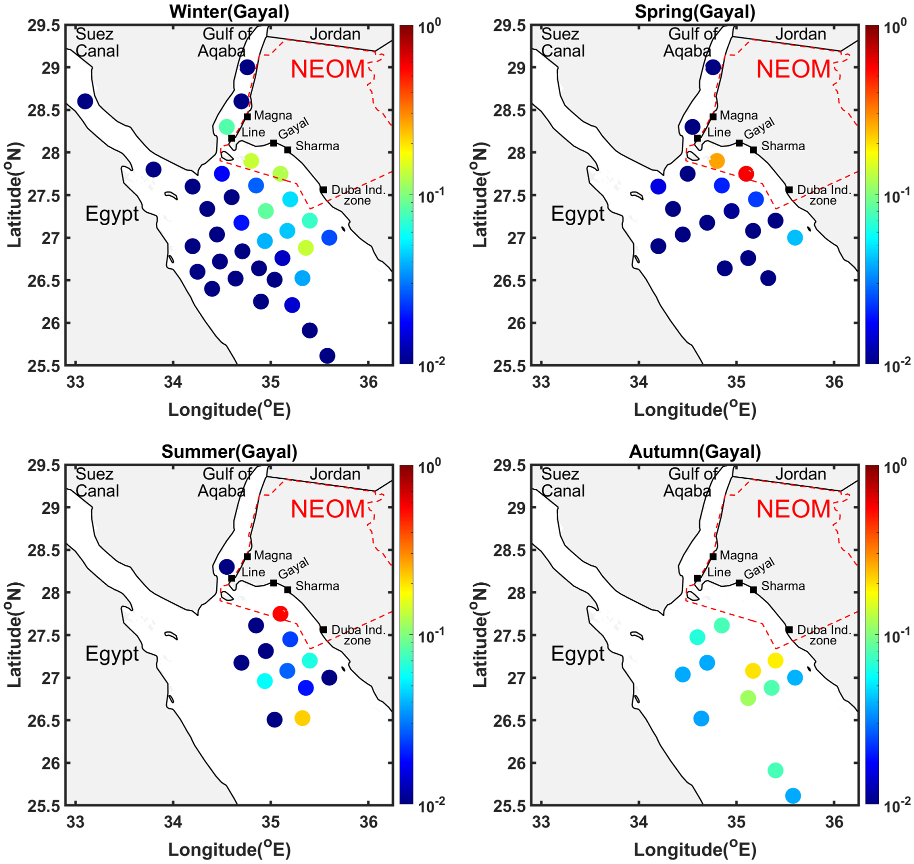

Supplementary Figure 12. Risk probabilities for shorelines of Gayal. The probabilities, estimated using Eq. 1, characterize the region of dependence of the overall risk. The plot title indicates the season in which the releases occur.
